# Hypothetical blood-pressure-lowering interventions and risk of stroke and dementia

**DOI:** 10.1007/s10654-020-00694-5

**Published:** 2020-11-27

**Authors:** Liliana Paloma Rojas-Saunero, Saima Hilal, Eleanor J. Murray, Roger W. Logan, Mohammad Arfan Ikram, Sonja A. Swanson

**Affiliations:** 1 Department of Epidemiology, Erasmus University Medical Center, PO Box 2040, 3000 CA Rotterdam, The Netherlands; 2 Department of Radiology and Nuclear Medicine, Erasmus University Medical Center, Rotterdam, The Netherlands; 3 Saw Swee Hock School of Public Health, National University of Singapore and National University Health System, Singapore, Singapore; 4 Department of Epidemiology, Harvard TH Chan School of Public Health, Boston, MA USA; 5 Department of Epidemiology, Boston University School of Public Health, Boston, MA USA

**Keywords:** Hypertension, Dementia, Stroke, Target trial, g-Formula

## Abstract

We aimed to study the effects of hypothetical interventions on systolic blood pressure (SBP) and smoking on risk of stroke and dementia using data from 15 years of follow-up in the Rotterdam Study. We used data from 4930 individuals, aged 55–80 years, with no prior history of stroke, dementia or cognitive impairment, followed for 15 years within the Rotterdam Study, a population-based cohort. We defined the following sustained interventions on SBP: (1) maintaining SBP below 120 mmHg, (2) maintaining SBP below 140 mmHg, (3) reducing SBP by 10% if above 140 mmHg, (4) reducing SBP by 20% if above 140 mmHg, and a combined intervention of quitting smoking with each of these SBP-lowering strategies. We considered incident stroke and incident dementia diagnoses as outcomes. We applied the parametric g-formula to adjust for baseline and time-varying confounding. The observed 15-year risk for stroke was 10.7%. Compared to no specified intervention (i.e., the “natural course”), all interventions that involved reducing SBP were associated with a stroke risk reduction of about 10% (e.g., reducing SBP by 20% if above 140 mmHg risk ratio: 0.89; 95% CI 0.76, 1). Jointly intervening on SBP and smoking status further decreased the risk of stroke (e.g., risk ratio: 0.83; 95% CI 0.71, 0.94). None of the specified interventions were associated with a substantive change in dementia risk. Our study suggests that a joint intervention on SBP and smoking cessation during later life may reduce stroke risk, while the potential for reducing dementia risk were not observed.

**Electronic supplementary material:**

The online version of this article (10.1007/s10654-020-00694-5) contains supplementary material, which is available to authorized users. A Spanish version of the manuscript is provided as supplementary material.

## Introduction

The increase in life expectancy over the past decades has profound implications on the occurrence of diseases. As a result of the rapid demographic aging, the burden from common age-related diseases such as stroke and dementia are expected to rise dramatically [1]. As such, effective strategies to prevent or delay the onset of such diseases are in dire need. Targeting generally healthy individuals for age-related chronic diseases has the potential to have the greatest overall impact on population health [2].

High blood pressure is a well-known modifiable risk factor for stroke [3]. It is also a proposed risk factor for dementia [4], though the specific biological mechanisms are heterogenous and less clear [5]. Randomized clinical trials have reported that treatment of hypertension reduces the risk of first-ever stroke by 35–40% among elderly patients with systolic hypertension [6, 7]. Some observational studies have assessed the association between lifestyle factors (i.e. unhealthy diet, smoking, drinking and physical inactivity) and stroke risk and have reported that 35–55% of stroke events were attributed to lifestyle factors [8–10]. Unlike for stroke, the evidence from trials and observational studies supporting the effects of lowering blood pressure on dementia risk is limited. Overall such trials did not have dementia as the primary outcome, were focused on highly selected patient groups and had short follow-up time (e.g., 2–4 years of follow-up) [6, 11–18]. In contrast, prior observational studies assessing the effect of systolic blood pressure or antihypertension medication were conducted in population-based cohorts with longer follow-up [19, 20]. However, in both settings, most studies were not conducted to estimate the effect of a sustained treatment strategy with appropriate account for time-varying confounding and attention to competing risk of death.

As a several-years-long randomized trial in the general population has not been conducted (and may be infeasible), decisions today regarding dynamic interventions on blood pressure and other lifestyle changes (e.g., quitting smoking) can be empirically informed using observational data to emulate a “target trial” [21–25]. Target trial emulation requires clear specification of the trial protocol elements and, when assessing interventions sustained over time, analytic methods known as “g-methods” are required to appropriately account for time-dependent confounding [26]. Previous studies have shown how results from observational studies can closely align with results from randomized controlled trials when the target trial framework is implemented [27, 28]. In this study, we emulate a target trial to estimate the sustained effects of several hypothetical interventions on systolic blood pressure (SBP) control, including in combination with an intervention on smoking over follow-up, on the risk of first-ever stroke and dementia using data from 15 years of follow-up in the Rotterdam Study.

## Methods

We begin by briefly describing the target trial specifications and then how we attempt to emulate the trial using data from the Rotterdam Study. A detailed comparison between the target trial and the emulation using observational data, is provided in the online resource, Table e-1.

### Target trial specification

#### Eligibility criteria:

Individuals 55–80 years old, with no prior history of stroke, transient ischemic attack, Parkinson´s Disease, Parkinsonism, dementia or cognitive impairment.

#### Treatment strategies:

Eligible individuals are assigned to one of the following sustained strategies, to be followed for the duration of the study: (1) maintaining SBP below 120 mmHg, (2) maintaining SBP below 140 mmHg, (3) reducing SBP by 10% if above 140 mmHg, (4) reducing SBP by 20% if above 140 mmHg. The means for following these strategies are not pre-specified (i.e., SBP may be reduced via lifestyle or medication interventions); we return to this point in the discussion. Given the known health effects of smoking, we further considered the intervention of (5) quitting smoking, and also four joint interventions combining (5) with (1), (2), (3), and (4). We compare all these strategies to the “natural course”, which represents no pre-specified treatment strategy. Of note, strategies (1) and (2) align with recent studied strategies in a randomized trial [17], while (3) and (4) perhaps are more achievable in practice.

#### Outcome recording:

The two primary outcomes of interest are first stroke event and dementia diagnosis within 15 years of follow-up, as recorded by continuous linkage to medical records and periodic cognitive assessments. Death preceding the outcome was treated as a competing event.

#### Start and end of follow-up:

Each eligible individual is followed from when they meet our eligibility criteria described above. They are followed until first stroke event, dementia diagnosis, death, incomplete follow-up, or administrative end of follow-up after 15 years from baseline.

### Target trial emulation

#### Study design and public involvement:

To emulate the described target trial, we used data from The Rotterdam Study (RS), a population-based prospective cohort study among middle age and elderly persons living in the Ommoord district in the city of Rotterdam, the Netherlands. Participants living in the district were invited to participate in the cohort between 1990 and 1993. All participants underwent questionnaire administration, physical and clinical examinations, and blood sample collection at baseline (1990–1993) and at follow-up visits from 1993–1995, 1997–1999, 2002–2005 [29].

#### Eligibility criteria:

**Fig. 1:**
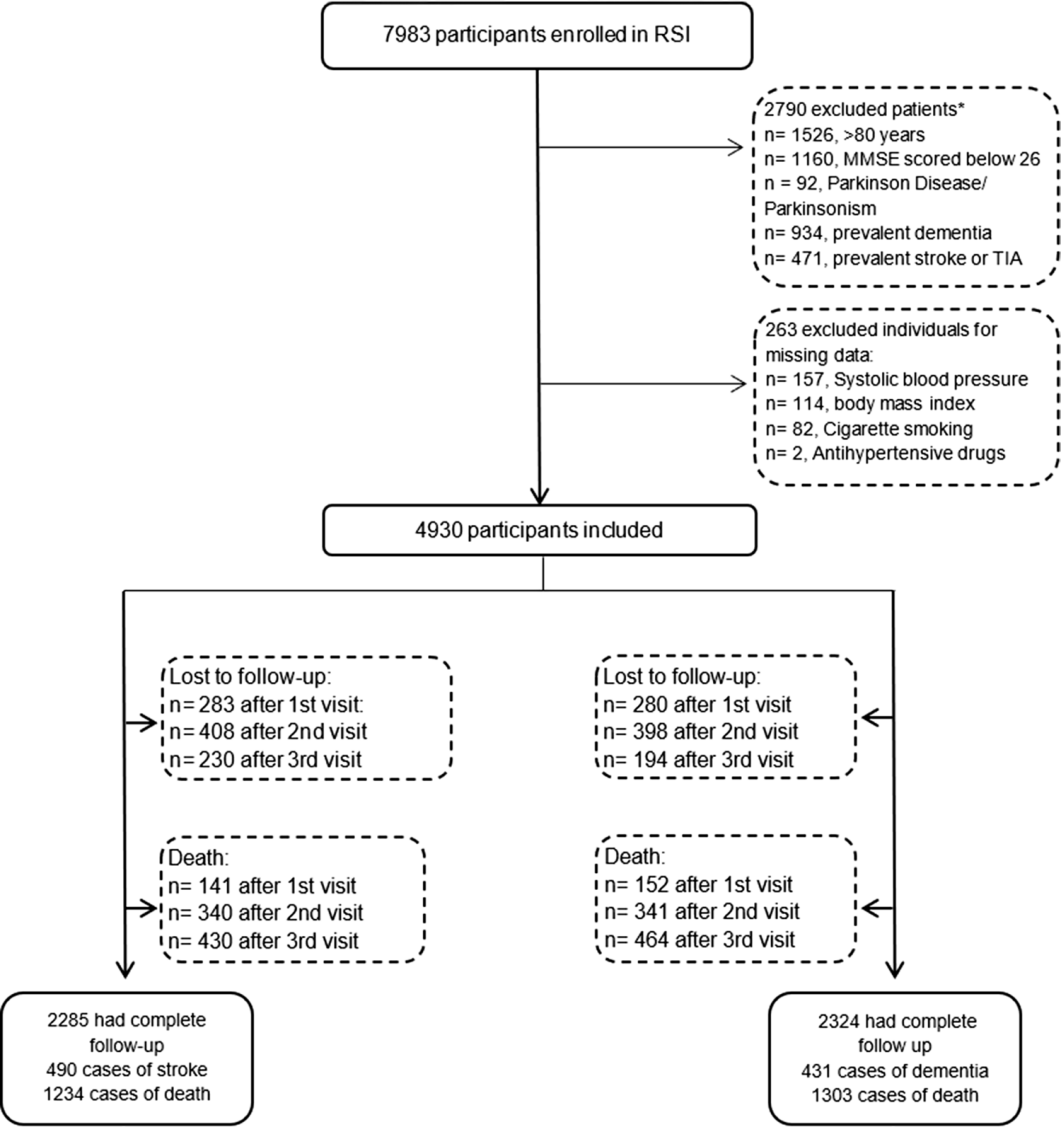
Flowchart

Same as specified above. Prior history of stroke, transient ischemic attack, Parkinson´s Disease, Parkinsonism and dementia were assessed by using home interviews and by reviewing medical records, and cognitive impairment defined as a Mini Mental State Examination (MMSE) below 26 at first study visit. Thus, of the 7983 persons who participated at baseline, 5193 were considered eligible for this study based on the above-mentioned criteria. We further required complete information at baseline on SBP, BMI, smoking status and hypertensive medication, giving a final sample size of 4930 participants (Fig. 1). Participants with missing covariates, which represent a 5% of the eligible sample, were, on average, three years older than those included, had a higher frequency of primary education and had a higher prevalence of heart disease and diabetes at baseline (Online resource, Table e-2).

#### Treatment strategies:

Same as specified above. SBP was measured in two readings using a random-zero sphygmomanometer in a sitting position, and the mean of both measurements was calculated during each follow-up visit. Smoking habit for cigarettes was collected using a detailed questionnaire and was categorized as never, current and former.

#### Outcome recordings: 

Incident stroke was collected by continuously monitoring through computerized linkage of the study database and digitized medical records from general practitioners and the Regional Institute for Outpatient Mental Health Care. For participants who moved outside the study district or lived in nursing homes, medical records were regularly checked by contacting their treating physicians. Research physicians reviewed all potential strokes using hospital discharge letters and information from general practitioners and nursing home physicians. An experienced vascular neurologist verified the stroke diagnosis [30, 31]. In accordance with World Health Organization criteria, stroke was defined as a syndrome of rapidly emerging clinical signs of focal or global disturbance of cerebral function. Symptoms should last ≥24 h or cause death, with no apparent cause other than of vascular origin.

Dementia diagnosis was collected by screening during the cohort visits, using MMSE and the Geriatric Mental Schedule (GMS) organic level. Screen-positives (MMSE < 26 or GMS organic level > 0) subsequently underwent an examination and informant interview with the Cambridge Examination for Mental Disorders in the Elderly. A consensus panel led by a consultant neurologist established the final diagnosis according to standard criteria for dementia (DSM-III-R). Additionally, participants were continuously followed up for the occurrence of dementia through automated linkage of general practitioners’ medical records with the study database [32, 33] as with stroke.

Vital status was obtained on a weekly basis via municipal population registries and through general practitioners’ and hospitals’ databases. All-cause mortality was defined as participants who died from any cause during the total follow-up period.

#### Start and end of follow-up:

We defined baseline as the date of recruitment in the Rotterdam Study for individuals for whom the above-described eligibility criteria were met on that date. Study participants were followed up from study baseline until stroke, dementia, death, censoring due to loss to follow-up, or 15 years after baseline, whichever occurred first. We defined loss to follow-up for stroke as follows: participants who skipped a visit or were lost to follow-up were censored at the last year in which the next visit could have taken place. Of the included participants who did not develop the main outcome or died during follow-up, 283 (9%) were lost after the first visit, 408 (13%) after the second visit, 230 (7%) after the third visit, and 2285 (71%) were censored after the fourth round. For dementia analyses, we followed participants until dementia diagnosis, death, or censored as previously defined. Of the 4930 included participants, 280 (9%) were lost after the first visit, 398 (12%) after the second visit, 194 (6%) after the third visit, and 2324 (73%) were censored after the fourth round (Fig. 1).

### Statistical analysis

To estimate the risk of stroke and dementia under the described hypothetical interventions, we used the parametric g-formula, an extension of standardization to time-varying exposures and confounders. Under the assumptions of no unmeasured confounding and no model misspecification, this method provides an estimate of the risk of outcomes under full adherence to different hypothetical sustained interventions [22, 25, 34–36].

The simplified steps for the parametric g-formula using stroke as the outcome are described as follows:

1. Fit parametric regression models for each of the time-varying covariates, as a function of baseline covariates and covariates history among participants followed up to time *k*.
2. Fit parametric regression models for stroke and death, as a function of baseline covariates and covariates history among participants followed up to the time *k*, using pooled logistic regression to approximate time-to-failure risk.
3. Use a Monte Carlo simulation to generate life histories for a pseudo-population of 10,000 simulated individuals.
  - Baseline covariates are randomly sampled with replacement from the original population.
  - The values of time-varying covariates are drawn from the parametric distribution in Step 1.
  - The value of the covariates that will be “intervened” on is set according to the defined strategy (skip this step for the “natural course strategy”).
  - The predicted risk of dementia and death is calculated for each individual in the pseudo-population.
4. Calculate the mean predicted risk of stroke and death at 15 years in the pseudo-population.
5. Calculate the risk difference between each strategy and the natural course.
6. Repeat previous steps in 500 bootstraps samples to obtain the 95% confidence interval (CI).
7. For each strategy repeat step 3–6.

The same steps were performed with dementia as an outcome. Our primary analyses consisted of models with baseline confounders which included: age with a quadratic term, sex, APOE-ε4 carrier, history of type-II diabetes mellitus, history of heart disease, education level, baseline SBP with a cubic term. Additionally, we also included time-varying covariates: the visit process, SBP, cholesterol, BMI, alcohol intake, smoking status, hypertensive medication, incident heart disease, incident diabetes, incident cancer, incident transient ischemic attack and incident Parkinson Disease or Parkinsonism. Details on measurements of these variables are available in the online supplementary material (Measurements). When stroke was the principal outcome, we included dementia as a time-varying confounder, and vice versa. All covariates that were measured during the visit process were modeled under the condition of having attended the visit (Online resource, Table e-3). To probe for potential model misspecification*,* we estimated the difference between the observed mean value and the predicted mean value for each covariate (Online resource, Figure e-1, Figure e-2). We also conducted a sensitivity analysis of reordering the time-varying covariates to probe potential model misspecification.

The results are presented as the average causal effect under each hypothetical intervention at 15 years of follow-up compared to the natural course as a risk ratio and risk difference. For each intervention, we further report the cumulative proportion of participants who would have had to have been intervened on during the follow-up, to adhere to the strategy. We additionally presented the standardized cumulative incidence curves, comparing the risk over time under the natural course and the joint treatment strategy.

#### Competing risk analysis

During follow-up, individuals can die before developing stroke and dementia, and interventions may affect the risk of death in our main analysis. For this reason, we estimate the risk of stroke and dementia taking into account that individuals can also progress to death. This means that the effect on our main outcome is through all pathways between the interventions and the outcome, including those possibly mediated by this competing event. We additionally run the primary analysis considering death as a censoring event, however the interpretation in this setting emulates a counterfactual world in which death could be entirely prevented, which would not be realistic and relies on additional stronger no-unmeasured-confounding assumptions [37]. Finally, we performed analysis considering the effect of each intervention in the composite outcome with death (i.e., stroke and death, dementia and death).

#### Subgroup analysis

We repeated our primary analyses within the following subgroups: age between 55 and 65 years; age between 66 and 80 years; women, men; without hypertensive medication at baseline; and free of heart disease at baseline.

All g-formula analyses were conducted using SAS 9.4 software and the GFORMULA macro that is publicly available at http://www.hsph.harvard.edu/causal/software. The SAS code for the GFORMULA macro call for our primary analysis is available on the following repository https://github.com/palolili23/ht_trial_gformula.

## Results

Table 1 shows the baseline characteristics of the study participants. The mean age of the participants was 66 years and 57% were women. The mean SBP at baseline was 137 mmHg, and 24% were current cigarette smokers.

**Table 1:** Characteristics of cohort at baseline (n = 4930)

	Overall
Female, n (%)	2824 (57.3)
Age, mean (SD) (years)	66.2 (6.6)
Apoe4 carrier, n (%)	
Not carrier	3388 (68.7)
Carrier	1322 (26.8)
Missing	220 (4.5)
Education level, n (%)	
Primary	2378 (48.6)
Further	2044 (41.8)
Higher	472 (9.6)
MMSE, mean (SD)	28.2 (1.2)
BMI, mean (SD) (kg/m^2^)	26.3 (3.6)
Cigarette smoking, n (%)	
Never	1551 (31.5)
Former	2193 (44.5)
Current	1186 (24.1)
Alcohol intake, mean (SD) (g/day)	10.7 (15.3)
Systolic blood pressure, mean (SD) (mmHg)	137.3 (21.5)
Total cholesterol, mean (SD) (mmol/dl)	6.7 (1.2)
HDL, mean (SD) (mmol/dl)	1.4 (0.4)
Prevalent hypertension, n (%)	2789 (56.6)
Hypertension medication, n (%)	1360 (27.6)
Prevalent heart disease, n (%)	368 (7.7)
Prevalent cancer, n (%)	20 (0.4)
Prevalent diabetes, n (%)	432 (12.9)

*MMSE* Mini Mental State Examination, *BMI* body mass index, *HDL* high density lipoproteins

### Stroke risk

**Fig. 2:**
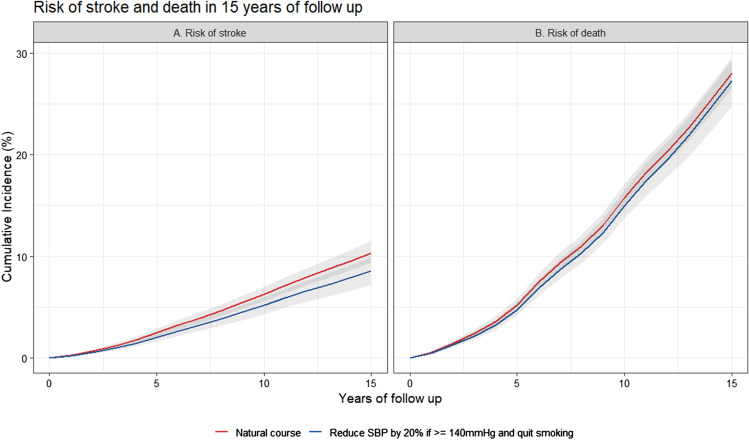
Risk of stroke and death under the natural course and under the joint intervention: Reduce SBP by 20% if above 140 mmHg and quit smoking at 15 years of follow-up

During the 15 years of follow-up, there were 490 cases of incident stroke and 1234 deaths. The observed 15 years risk for stroke was 10.3% and under the simulated natural course was 10.3% (95%CI 9.3, 11.5). The risk of stroke under the different hypothetical treatment strategies are presented in Table 2. Overall, all interventions that lowered SBP under a threshold reduced the risk of stroke by approximately 10% compared to the natural course during the study period. Although all interventions on SBP had a similar association with risk of stroke, the most intensive treatment strategy studied (“maintaining SBP below 120 mmHg”) required intervening in 98% of the population at some point in follow-up, which involved 15% more people compared to all other strategies. By contrast, smoking cessation was associated with a reduction in stroke risk by 7% (RR 95%CI 0.89, 0.97) compared to the natural course, and required an intervention on only 26% of the population. All joint interventions showed a larger reduction in the risk of stroke. For example, lowering SBP by 20% if above 140 mmHg and quitting smoking was associated with a 17% (RR 95% CI 0.71, 0.94) reduction in the risk of stroke over the study period compared to the natural course as observed in Fig. 2A.

**Table 2:** Risk of stroke at 15 years of follow-up under natural course and hypothetical interventions

No.	Intervention	Absolute risk (95% CI)	Risk ratio (95% CI)	Risk difference (95% CI)	Total intervened (%)
0	Natural course	10.3 (9.3, 11.5)	-	-	0.00
1	Maintaining SBP below 120 mmHg	9.0 (7.5, 10.7)	0.87 (0.74, 1.02)	−1.3 (−2.8, 0.2)	97.8
2	Maintaining SBP below 140 mmHg	9.3 (8.2, 10.6)	0.90 (0.83, 0.98)	−1.0 (−1.8, −0.2)	83.5
3	Reducing SBP by 10% if above 140 mmHg	9.3 (8, 10.6)	0.90 (0.80, 0.98)	−1.1 (−2.1, −0.2)	82.7
4	Reducing SBP by 20% if above 140 mmHg	9.2 (7.8, 10.6)	0.89 (0.76, 1.00)	−1.1 (−2.5, 0.0)	82.7
5	Quitting smoking	9.6 (8.6, 10.8)	0.93 (0.89, 0.97)	−0.7 (−1.2, −0.3)	25.9
6	Joint 1 + 5	8.3 (6.9, 10.0)	0.81 (0.68, 0.95)	−2.0 (−3.4, −0.5)	98.7
7	Joint 2 + 5	8.8 (7.6, 9.9)	0.85 (0.76, 0.92)	−1.6 (−2.6, −0.8)	88.6
8	Joint 3 + 5	8.6 (7.4, 9.8)	0.83 (0.74, 0.92)	−1.7 (−2.8, −0.8)	88.2
9	Joint 4 + 5	8.5 (7.2, 9.9)	0.83 (0.71, 0.94)	−1.8 (−3.1, −0.6)	88.2

Estimates were based using the parametric g-formula with fixed covariates: age, sex, education, systolic blood pressure, history of diabetes and history of heart disease at baseline; and time-varying covariates: visit process, smoking status, systolic blood pressure, body mass index, hypertension medication, total cholesterol and diagnosis of diabetes, heart disease, Parkinson disease, Parkinsonism, transient ischemic attack, dementia or cancer

SBP: Systolic blood pressure (mmHg)

### Dementia risk

**Fig. 3:**
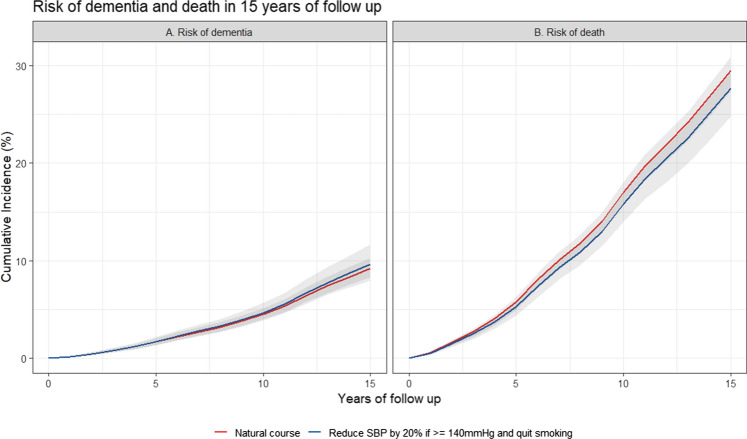
Risk of dementia and death under the natural course and under the joint intervention: Reduce SBP by 20% if above 140 mmHg and quit smoking at 15 years of follow-up

During the 15 years of follow-up, there were 431 cases of dementia and 1303 deaths. The observed 15-year risk for dementia was 8.9% and under the simulated natural course was 9.2% (95% CI 8.2, 10.3%). The risk of dementia under the different hypothetical interventions are shown in Table 3. Overall, none of the treatment strategies involving SBP were associated with substantial changes in risk of dementia. For example, the treatment strategy “maintaining SBP below 120 mmHg” was associated with a 6% (RR 95% CI 0.90, 1.24) increase in dementia risk compared to the natural course. This pattern was likewise seen for the treatment strategy of smoking cessation and joint treatment strategies involving lowering SBP and smoking cessation. For example, lowering SBP by 20% if above 140 mmHg and quit smoking was associated with an increment in dementia risk of 5% (RR 95% CI 0.92, 1.20) as observed in Fig. 3A.

**Table 3:** Risk of dementia at 15 years of follow-up under natural course and hypothetical interventions

No.	Intervention	Absolute risk (95% CI)	Risk ratio (95% CI)	Risk difference (95% CI)	Total intervened (%)
0	Natural course	9.2 (8.2, 10.3)	-	-	0.0
1	Maintaining SBP below 120 mmHg	9.7 (8, 11.9)	1.06 (0.90, 1.24)	0.6 (−0.9, 2.2)	98.2
2	Maintaining SBP below 140 mmHg	9.2 (8, 10.7)	1.01 (0.92, 1.09)	0.1 (−0.7, 0.8)	83.0
3	Reducing SBP by 10% if above 140 mmHg	9.2 (8, 10.9)	1.01 (0.92, 1.11)	0.0 (−0.8, 1.0)	83.3
4	Reducing SBP by 20% if above 140 mmHg	9.5 (8, 11.4)	1.04 (0.91, 1.18)	0.3 (−0.8, 1.7)	83.3
5	Quitting smoking	9.3 (8.4, 10.6)	1.01 (0.98, 1.06)	0.1 (−0.2, 0.5)	25.9
6	Joint 1 + 5	9.9 (8, 12.2)	1.08 (0.92, 1.26)	0.8 (−0.8, 2.4)	98.8
7	Joint 2 + 5	9.3 (8.1, 10.9)	1.02 (0.93, 1.12)	0.2 (−0.6, 1.1)	88.2
8	Joint 3 + 5	9.3 (8, 11.1)	1.02 (0.93, 1.14)	0.2 (−0.7, 1.3)	88.6
9	Joint 4 + 5	9.6 (8, 11.6)	1.05 (0.92, 1.20)	0.4 (−0.7, 2.0)	88.6

Estimates were based using the parametric g-formula with fixed covariates: age, sex, education, systolic blood pressure, history of diabetes and history of heart disease at baseline; and time-varying covariates: visit process, smoking status, systolic blood pressure, body mass index, hypertension medication, total cholesterol and diagnosis of diabetes, heart disease, Parkinson disease, Parkinsonism, transient ischemic attack, dementia or cancer

*SBP* systolic blood pressure (mmHg)

### Alternative analyses for competing event

Given that death was modeled as a competing event for both outcomes (stroke and dementia), we present the effect of the intervention “lowering 20% of SBP if above 140 mmHg and quit smoking” on the risk of death in Figs. 2B and  3B respectively. Treating death as a censoring event and as part of a composite outcome did not meaningfully change the conclusions, as presented in the online resource, Table e-4, e-5, e-12 e-13.

### Subgroup analyses

Table 4 provides estimates for the treatment strategy “lowering 20% of SBP if above 140 mmHg and quit smoking” on the risk of stroke and dementia by subgroups (age, sex, without hypertension medication at baseline, free of heart disease at baseline) compared to the natural course. Estimates were relatively consistent for stroke risk across subgroups, with the exception of individuals with age below 65 years among whom there appeared to be a much stronger association (RR: 0.75, 95% CI 0.56, 0.98).For dementia risk, subgroup analyses present similar findings. Additional treatment strategies are presented in the online resource, Table e-6 to e- 11, e-14 to e-19.

**Table 4:** Subgroup analyses comparing the joint intervention of reducing SBP by 20% if above 140mmHg and quitting smoking to the natural course

Subgroup	Stroke				Dementia			
	Risk under natural course (95% CI)	Risk under joint intervention (95% CI)	Risk ratio (95% CI)	Risk difference (95% CI)	Risk under natural course (95% CI)	Risk under joint intervention (95% CI)	Risk ratio (95% CI)	Risk difference (95% CI)
Complete cohort	10.3 (9.3, 11.5)	8.5 (7.2, 9.9)	0.83 (0.71, 0.94)	−1.8 (−3.1, −0.6)	9.2 (8.2, 10.3)	9.6 (8.0, 11.6)	1.05 (0.92, 1.20)	0.4 (−0.7, 2.0)
Age below 65 (n = 2303)	6.3 (5.0, 7.9)	4.7 (3.3, 6.4)	0.75 (0.56, 0.98)	−1.6 (−2.7, −0.2)	4.0 (3.2, 5.7)	4.2 (3.0, 6.1)	1.04 (0.73, 1.37)	0.2 (−1.0, 1.4)
Age between 65 and 80 (n = 2627)	14.0 (12.6, 15.8)	11.9 (9.6, 14.5)	0.85 (0.71, 1.00)	−2.2 (−4.3, 0.0)	13.8 (12.4, 15.6)	14.2 (12.0, 17.3)	1.03 (0.87, 1.20)	0.4 (−1.8, 2.9)
Women (n = 2824)	9.3 (8.1, 10.9)	7.7 (6.1, 10.3)	0.83 (0.71, 1.02)	−1.6 (−2.9, 0.3)	11.2 (9.7, 12.7)	10.1 (8.4, 12.3)	0.91 (0.73, 1.05)	−1.1 (−3.4, 0.5)
Men (n = 2106)	11.6 (10.2, 13.5)	9.4 (7.5, 12.3)	0.81 (0.67, 1.01)	−2.2 (−4.0, 0.1)	7.0 (6.0, 8.9)	9.8 (7.2, 13.0)	1.40 (1.12, 1.64)	2.8 (0.8, 4.5)
Without hypertension medication at baseline (n = 3570)	9.0 (8.1, 10.6)	7.0 (5.8, 9.1)	0.78 (0.66, 0.94)	−2.0 (−3.3, −0.5)	8.8 (8.0, 10.2)	9.9 (7.7, 11.8)	1.12 (0.95, 1.27)	1.1 (−0.4, 2.4)
Free of heart disease at baseline (n = 4406)	9.9 (9.1, 10.9)	8.2 (6.8, 10.1)	0.83 (0.70, 0.99)	−1.6 (−2.9, −0.1)	8.8 (8.0, 10.0)	9.2 (7.6, 11.1)	1.05 (0.9, 1.16)	0.4 (−0.9, 1.6)

*SBP* systolic blood pressure (mmHg)

Estimates were based using the parametric g-formula with fixed covariates: age, sex, education, systolic blood pressure, history of diabetes and history of heart disease at baseline; and time-varying covariates: visit process, smoking status, systolic blood pressure, body mass index, hypertension medication, total cholesterol and diagnosis of diabetes, heart disease, Parkinson disease, Parkinsonism, transient ischemic attack, dementia or cancer

## Discussion

Our study suggests that intervening on blood pressure could reduce stroke risk by approximately 10% over 15 years of follow-up in a population-based setting, and that combining such interventions with smoking cessation could result in an overall reduction of 18%. In contrast, our study is consistent with these same interventions having null or opposite effects on risk of dementia, taking into account that these estimates could be affected by how the interventions may decrease the risk of death.

Our results on stroke risk are comparable in direction but not quite in magnitude of prior studies’ effect estimates. A previous meta-analysis of randomized trials has shown that a 10 mmHg reduction in blood pressure decreases the risk of stroke by 27%, though this effect was observed in a high-risk population of individuals with cardiovascular diseases [38]. The most comparable observational study to ours was one emulating hypothetical treatment strategies to reduce SBP in middle-aged (i.e., baseline mean age 46.1 years or about 20 years younger that our study) healthy individuals from Norway, also using the g-formula. This study showed that a 23 mmHg average reduction in blood pressure of 120 mmHg and above resulted in a 45% reduction in stroke risk over a 15 year period [35]. The difference in the risk estimates might be due to differences in the average age of the study populations. Of note, no differences in proportional risk reductions were reported in trials involving persons with systolic blood pressure < 130 mmHg and those at high risk (≥160 mmHg) [38]. Our study also adds to these prior studies by considering a joint intervention with smoking cessation, although our combined strategies appears to reduce stroke risk by a much lower amount (18%) than those reported previously in observational studies (35–55%) [8–10].

To compare the results from our hypothetical intervention on blood pressure (dropping below 120 or 140 mmHg over time) in dementia risk with previous research, we must consider the differences in the eligibility criteria, treatment strategies and analytical choices. Our target trial by design follows similar treatment strategies such as the Systolic Blood Pressure Intervention Trial (SPRINT) MIND trial, however this trial considered eligible individuals as those who had risk of cardiovascular disease [17], which represents a small subgroup of our population-based cohort. (Specifically, at most 290 individuals in the Rotterdam Study would meet criteria at baseline, based on our assessments of SBP, presence of cardiovascular disease other than stroke and history of diabetes.) Similarly, since previous trials were primarily designed to assess the effect of antihypertensive medication on the risk of stroke and did not have dementia as their primary outcome, they were tailored to a specific subgroup of individuals who required treatment. Eligibility criteria in other trials included having had a history of stroke, being above 80 years old and having a SBP above 160 mmHg [6, 11–18]. Furthermore, assessing the comparability of our findings with prior observational studies requires caution. A recent meta-analysis by Ding et al. studied the effect of taking any antihypertensive medication and specific antihypertensive medications and the risk of dementia stratified by SBP including five large population-based cohort [19]. However, they studied prevalent treatment use at baseline and only included baseline covariates for adjustment. Similarly, Walkert al. stratified individuals by longitudinal blood pressure patterns (mid-life and late-life normotension/hypertension), but covariates were measured during two of the six visits [39]. In contrast, we assessed the sustained effect of lowering SBP over time given time-updated covariates. Last, we consider that our findings reflect the relevance of the competing event of death by other causes, and how estimates are likely affected by the effect of interventions on the risk of death, and give a more comprehensive view of the implications of these results. This will be especially important when we stratify by characteristics that have a different survival distribution, as we observe in the different direction of the effects among women vs. men [40]. Reconsidering these points as part of how we frame research question and analytical decisions when using observational data will have a direct impact on results interpretation and clinical translation.

By leveraging a rich dataset in a population-based observational study, high-quality and frequent assessments of outcomes and key covariates, and the use of the parametric g-formula to account for the complex confounding structure assumed, we emulated a “target trial” that may be of key public health interest but would not be easily conducted as a randomized trial. However, like any observational data analysis, several assumptions need to be carefully weighed. The possibility of unmeasured confounding remains, and in particular we were unable to adjust for covariates such as types of antihypertension medications, LDL (as separate from total cholesterol), glucose and frailty in our analysis. Likewise, we used MMSE as a screening tool and excluded individuals with Parkinson disease or Parkinsonism symptoms at baseline, but it is possible that persons with subclinical cognitive impairment might be included in our analysis at baseline or during follow-up [41]. Self-reported smoking status is also subject to measurement error, although we did assess the consistency of our measurements over time. In addition, the parametric g-formula relies on several strong modeling assumptions. As reported in the online resource Figure e-1, Figure e-2, we observed an agreement between the mean estimated values of each variable (outcomes and covariates) under the natural course with their observed values, which supports but does not prove these model specifications hold [22]. Furthermore, we did not assess the effect of the hypothetical interventions in the specific clinical phenotypes of each disease, since numbers on the disaggregated clinical subtypes are small and is further complicated by each subtype being a competing event for each other.

Finally, another key point to reflect upon in interpreting our specified treatment strategies is that we did not, in fact, specify how SBP would be lowered. This means our estimates are based on the consistency assumption that lowering SBP through any available means (e.g., dietary changes, medication use, other lifestyle changes) would have the same effect on stroke or dementia risk, or otherwise are at best interpretable as estimates for an effect of a weighted average of several SBP-lowering strategies with weights determined by the frequency that the particular strategies occur in our specific population [42, 43]. Future studies that have more detailed assessments of these various SBP-lowering treatments are needed to disentangle treatment variation relevance and build upon this initial study. Furthermore, smoking cessation is one of several more specific behavioral interventions that could be assessed, as well as other metabolic factors described in current guidelines [3, 4]. Implementing the target-trial framework and defining research questions to study more refined or further specified treatment strategies is a crucial next step. Doing so will require rich, longitudinal data on the specific interventions under study; the level of specificity in the research questions that can be studied is hampered in part by the data currently available. Thus, while there are certainly limitations in terms of ambiguity to the interventions studied in the current paper, they represent an improvement (in terms of clarity and for informing decision-making) over etiologic studies that address SBP’s effects with a simplified version of the complexity of real data, and a step toward the types of interventions we may consider in practice.

Given previous considerations, studying population-level interventions as done here is particularly suitable for public health research, in that we can better understand how particular recommendations may affect stroke or dementia risk at the population-level rather than as estimated in high-risk subpopulations. Importantly, while the possible effect of blood pressure control on dementia risk remains debated, our findings nonetheless align with the recent WHO Report’s recommendation that lowering blood pressure has substantial benefits (in terms of stroke risk and mortality, here) that may motivate blood pressure control regardless of its possible effects on dementia risk [44].

## Electronic supplementary material

Below is the link to the electronic supplementary material.

Supplementary file1 (PDF 373 kb)

Supplementary file2 (PDF 322 kb)

## Data Availability

Data can be obtained on request. Requests should be directed toward the management team of the Rotterdam Study (secretariat.epi@erasmusmc.nl), which has a protocol for approving data requests. Because of restrictions based on privacy regulations and informed consent of the participants, data cannot be made freely available in a public repository.

## Code availability

The SAS code for the GFORMULA macro call for our primary analysis is available on the following repository https://github.com/palolili23/ht_trial_gformula.
