# Supplementary material for: Hypothetical blood-pressure-lowering interventions and risk of stroke and dementia
Source: Eur J Epidemiol. 2020 Nov 27;36(1):69–79. doi: 10.1007/s10654-020-00694-5 (PMC7847439; doi:10.1007/s10654-020-00694-5)
Supplement: Supplementary file 1 — Supplementary file1 (PDF 373 kb) [file 10654_2020_694_MOESM1_ESM.pdf]

Hypothetical blood-pressure-lowering interventions and risk of stroke and dementia

Online Resources

Authors: L. Paloma Rojas-Saunero, MD[1*]; Saima Hilal PhD[1,2,3*]; Eleanor J. Murray Sc.D [4,5], Roger W. Logan PhD [4]; M. Arfan Ikram PhD [1]; Sonja A. Swanson, Sc.D[1,4]

1. Department of Epidemiology, Erasmus University Medical Center, Rotterdam , the Netherlands

2. Department of Radiology and Nuclear Medicine, Erasmus University Medical Center, Rotterdam, the Netherlands

3. Saw Swee Hock School of Public Health, National University of Singapore, Singapore

4. Department of Epidemiology, Harvard TH Chan School of Public Health, Boston, MA, USA

5. Department of Epidemiology, Boston University School of Public Health, Boston, MA, USA

* These authors contributed equally to the manuscript

Correspondence:
L. Paloma Rojas-Saunero MD,
Department of Epidemiology, Erasmus MC University Medical Center
Dr.Molewaterplein 40, 3015 GD Rotterdam, The Netherlands. P.O. Box 2040
E-mail address l.rojassaunero@erasmusmc.nl,
Telephone number 010 704 34 88

Table e-1: Target trial description

Emulation of a target trial of multiple and joint strategies to lower the risk of stroke among adults from 55 to 80 years old.

Section	Target trial	Emulation using observation data
Aim	To estimate the effect of joint interventions on 15-year risk of stroke among people at risk	Same
Eligibility criteria	Individuals below 80 years old, with no prior history of stroke or transient ischemic attack, cognitive impairment, dementia diagnosis or Parkinson's disease or Parkinsonism.	Same + MMSE above 26 at baseline.
Treatment strategies	1. Maintaining SBP below 120 mmHg<br>2. Maintaining SBP below 140 mmHg<br>3. Reducing SBP by 10% if above 140 mmHg<br>4. Reducing SBP by 20% if above 140 mmHg<br>5. Quitting smoking<br>6. Joint 1 + 5<br>7. Joint 2 + 5<br>8. Joint 3 + 5<br>9. Joint 4 + 5.	Same
Comparison arm	Natural course	Same
Follow-up	Starts at first visit, ends after stroke diagnosis, death, lost to follow up or after 15 years since baseline, which ever happens first.<br>Annual checkups during trial to assess adherence and adverse effects.	Same + simulate visit process
Outcome	Stroke (Death as competing risk)	Same
Causal contrast of interest	What would have been observed if all individuals adhered to their assigned strategy over the 15 years of follow-up (Per protocol effect)	Same
Statistical analysis	Comparison of 15 year-risk of stroke between groups that received each treatment strategy, adjusted for post-baseline confounders associated with adherence to the treatment strategies and lost to follow-up	Same as per-protocol effect + adjustment of baseline

Measurements.

The information on covariates were also collected during each visit through several questionnaires, physical examination and blood samples. From each visit, we selected the following covariates: age, sex, education attained, BMI, Apolipoprotein E (APOE)- ε4 carrier status, alcohol intake (grams per day), cholesterol (mmol/dl) and hypertensive medication. Education attained was divided in three categories: 1) primary; 2) further: lower or intermediate general or vocational education, or higher general education; 3) higher: vocational education or university[1]. BMI was computed by dividing the weight in kg by the square of the height in meters. APOE genotype was determined using polymerase chain reaction on coded DNA samples[28]. Distribution of APOE genotype and allele frequencies was in the Hardy-Weinberg equilibrium. APOE-ε4 carrier status was defined by the presence of at least one ε4 allele. Alcohol intake was collected from a validated semiquantitative food-frequency questionnaire and units were harmonized to grams/day, based on the assumption that one unit (glass) of alcoholic beverage equals 10 grams of alcohol[2]. Hypertensive medication was categorized by the World Health Organization Anatomical Therapeutic Chemical (WHO ATC) classification as antihypertensives (c02), diuretics (c03), beta blockers (c07), calcium channel blockers (c08), and renin-angiotensin-aldosterone system modifying agents (c09). History of heart disease and diabetes was collected at baseline. Heart disease was defined as the history of myocardial infarction, atrial fibrillation and cardiac intervention such as angioplasty, coronary artery bypass grafting and other coronary revascularization procedures[3].

Additionally, the occurrence of incident heart diseases and cardiac interventions, incident diabetes, transient ischemic attack, Parkinson disease, Parkinsonism, and cancer diagnosis were recorded independent from the visit process. The specific date of diagnosis was obtained through an automated follow-up system that integrates data from different sources as was performed for stroke and dementia.

1. Ott, A. *et al.* Education and the incidence of dementia in a large population-based study: The Rotterdam Study. *Neurology* **52**, 663 LP-663 (1999).
2. Ruitenberg, A. *et al.* Alcohol consumption and risk of dementia: The Rotterdam Study. *Lancet* **359**, 281–286 (2002).
3. Leening, M. J. G. *et al.* Methods of data collection and definitions of cardiac outcomes in the Rotterdam Study. *Eur. J. Epidemiol.* (2012). doi:10.1007/s10654-012-9668-8

Table e-2. Characteristics of included and excluded (missing covariates) participants

Characteristics	Included (n = 4930)	Excluded (n = 263)
Age in years, mean (SD)	66.23 (6.6)	69.00 (6.9)
Women (%)	2824 (57.3)	155 (58.9)
Apoe 4 carrier	1322 (28.1)	59 (30.7)
Education		
Primary	2378 (48.6)	122 (56.5)
Further	2044 (41.8)	78 (36.1)
Higher	472 (9.6)	16 (7.4)
Prevalent heart disease	368 (7.7)	22 (13.3)
Prevalent diabetes	432 (12.9)	16 (16.3)
Prevalent cancer	20 (0.4)	1 (0.4)

Table e-3

Time-fixed covariates	Assessed	Functional form when used as predictor	Categories
Age at baseline	1st visit		
Education at baseline	1st visit	4 categories	Higher (4)<br>Further (3)<br>Primary (2)<br>Missing (1)
Sex	1st visit	2 categories	Female, male
Apoe 4 carrier	1st visit	3 categories	Non carrier (3)<br>Carrier (2)<br>Missing (1)
History of diabetes at baseline	1st visit	Indicator	Yes/No
History of heart disease diagnosis at baseline	1st visit	Indicator	Yes/No
**Time-varying covariates**	**Assessed**	**Functional form when used as predictor**	**Categories**
Second visit	Between year 1 and year 5	Indicator and time since switch	N/A
Third visit	Between year 5 and year 9 and only if visit 2 happened	Indicator and time since switch	N/A
Fourth visit	Between year 5 and year 14 and only if visit 3 happened	Indicator and time since switch	N/A
Smoking status	1st - 4th visit	3 categories	Current (3)<br>Former (2)<br>Never (1)
Alcohol intake (g/day)	1st - 4th visit	5 categories	>= 10 g/day (5)<br>5 - 10 g/day (4)<br>1 - 5 (3)<br>< 1 (2)<br>Missing (1)
Systolic blood pressure (mmHg)	1st - 4th visit	Continuous	
Body mass index	1st - 4th visit	Continuous	Splines in 18.5 20 25 30
Hypertension medication	1st - 4th visit	Indicator	Yes/No
Total Cholesterol	1st visit<br>3rd visit<br>4th visit	4 Categories	is.na(chol) ~ 1,<br><= 5.17 ~ 2,<br>> 5.17 & <6.21 ~3<br>>= 6.21 ~ 4
Diagnosis of diabetes type 2	Year of diagnosis	Indicator and time since switch	N/A
Diagnosis of heart disease	Year of diagnosis	Indicator and time since switch	N/A
Diagnosis of Parkinson's Disease or Parkinsonism	Year of diagnosis	Indicator and time since switch	N/A
Diagnosis of transient ischemic attack	Year of diagnosis	Indicator and time since switch	N/A
Diagnosis of stroke	Year of diagnosis	Indicator and time since switch	N/A
Diagnosis of cancer	Year of diagnosis	Indicator and time since switch	N/A
Diagnosis of dementia	Year of diagnosis	Indicator and time since switch	N/A

Figure e-1.

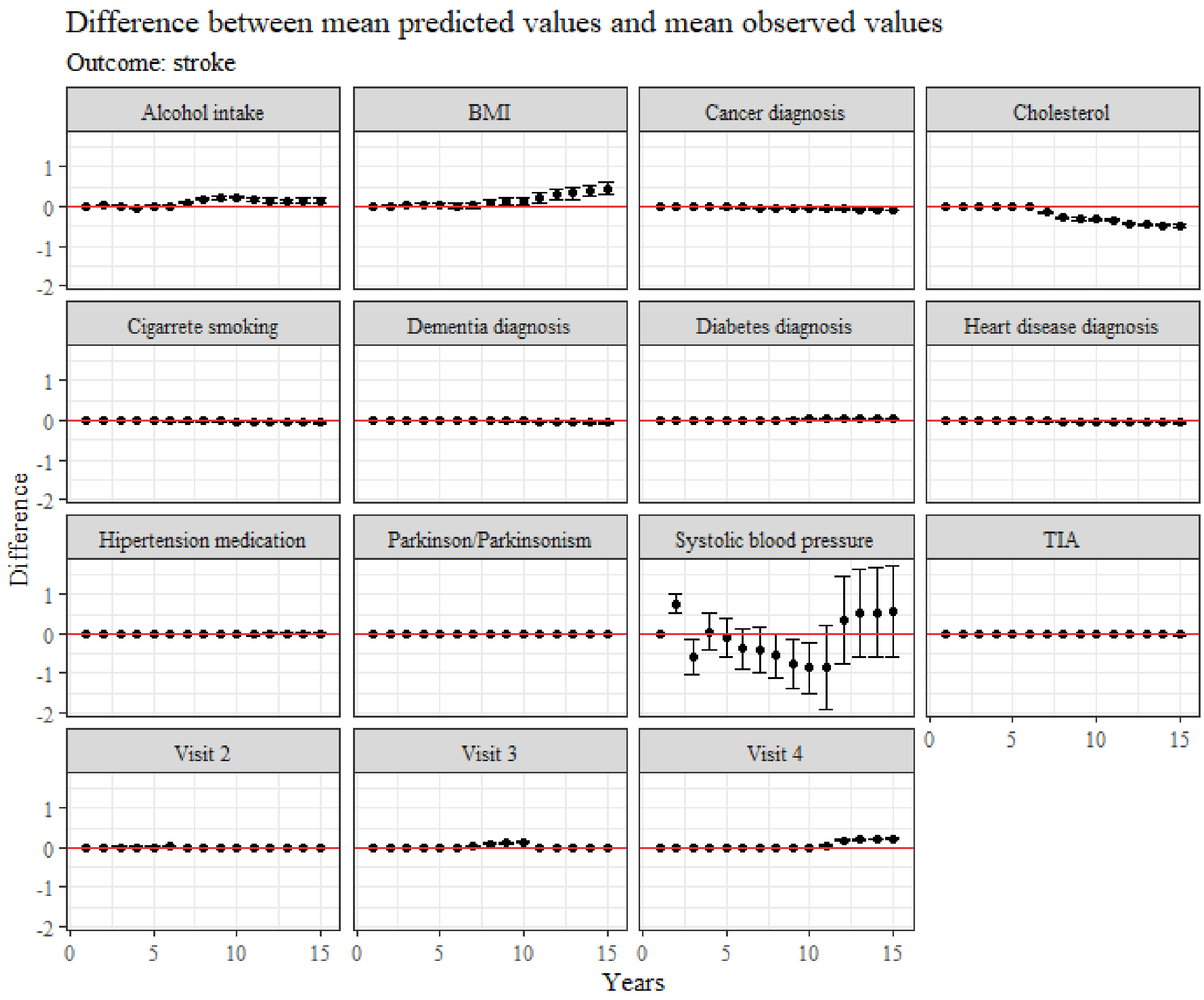

Difference between mean predicted values and mean observed values
Outcome: stroke
Alcohol intake
BMI
Cancer diagnosis
Cholesterol
Cigarrete smoking
Dementia diagnosis
Diabetes diagnosis
Heart disease diagnosis
Hipertension medication
Parkinson/Parkinsonism
Systolic blood pressure
TIA
Visit 2
Visit 3
Visit 4
Difference
Years
1
0
-1
-2
0
5
10
15

Figure e-2

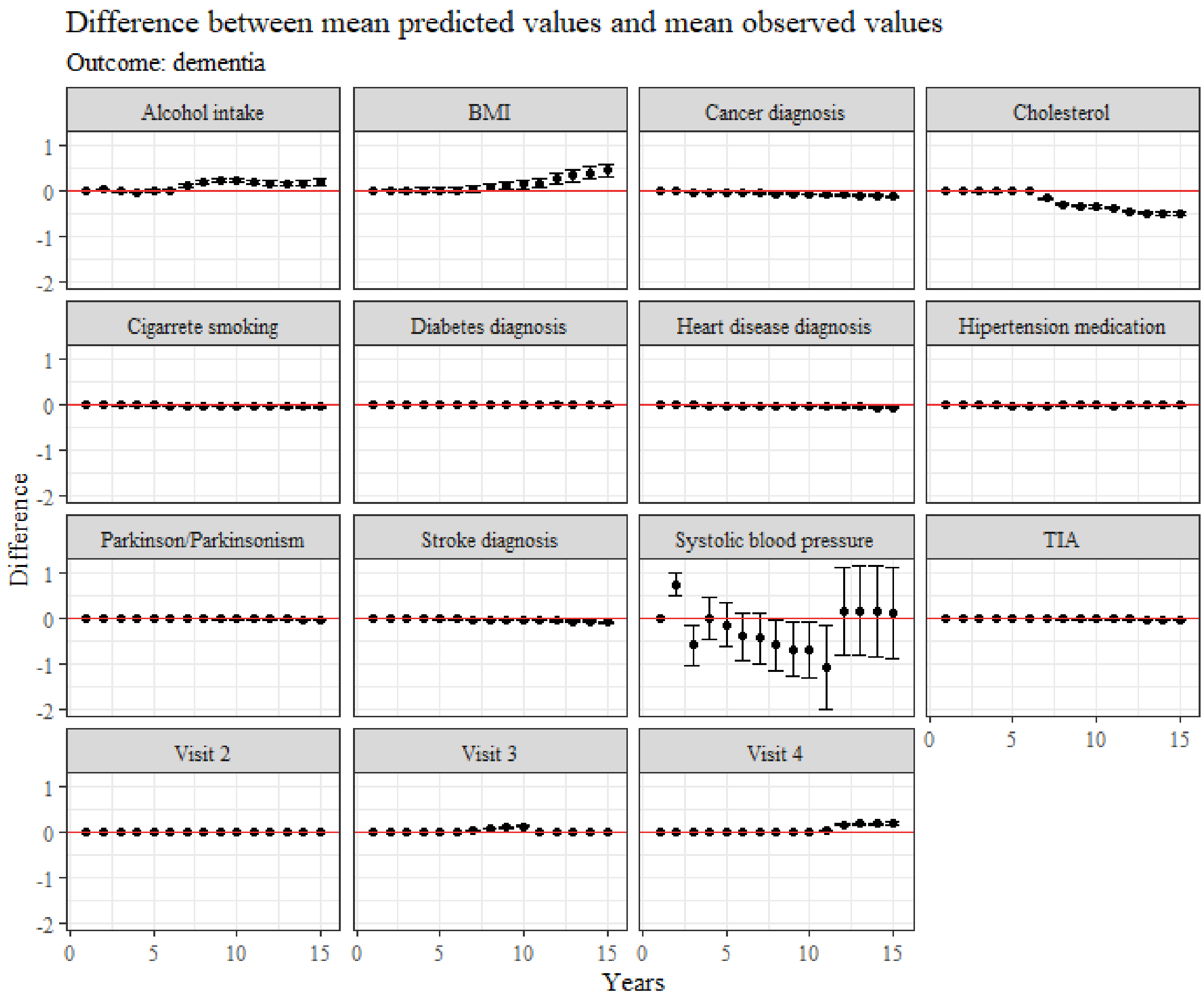
Difference between mean predicted values and mean observed values
Outcome: dementia
Alcohol intake
BMI
Cancer diagnosis
Cholesterol
Cigarrete smoking
Diabetes diagnosis
Heart disease diagnosis
Hipertension medication
Parkinson/Parkinsonism
Stroke diagnosis
Systolic blood pressure
TIA
Visit 2
Visit 3
Visit 4
Difference
Years
1
0
-1
-2
0
5
10
15

Table e-4

1. Stroke

1.1. Death as censored.
The observed risk at 15 years is **12.0%** .

No	Intervention	Absolute Risk (%) CI(95%)	Risk Ratio CI(95%)	Risk Difference CI(95%)	Total Intervened (%)
0	Natural course	14 (12.4, 16.1)	1 (1, 1)	0 (0, 0)	0.0
1	Maintaining SBP below 120 mmHg	12.6 (10.1, 15.3)	0.9 (0.75, 1.03)	-1.4 (-3.5, 0.4)	97.8
2	Maintaining SBP below 140 mmHg	12.7 (11, 14.8)	0.91 (0.83, 0.98)	-1.3 (-2.4, -0.3)	83.5
3	Reducing SBP by 10% if above 140 mmHg	12.7 (10.8, 14.8)	0.91 (0.82, 0.99)	-1.3 (-2.6, -0.2)	82.7
4	Reducing SBP by 20% if above 140 mmHg	12.7 (10.4, 15)	0.91 (0.78, 1.01)	-1.3 (-3, 0.1)	82.7
5	Quitting smoking	12.7 (11.2, 14.6)	0.9 (0.86, 0.94)	-1.4 (-2, -0.8)	25.9
6	Joint 1 + 5	11.3 (9.1, 13.7)	0.8 (0.67, 0.93)	-2.8 (-4.7, -0.9)	98.7
7	Joint 2 + 5	11.6 (9.9, 13.4)	0.83 (0.74, 0.9)	-2.4 (-3.8, -1.5)	88.6
8	Joint 3 + 5	11.4 (9.6, 13.3)	0.81 (0.72, 0.9)	-2.6 (-4, -1.4)	88.2
9	Joint 4 + 5	11.5 (9.3, 13.4)	0.82 (0.7, 0.92)	-2.6 (-4.3, -1.1)	88.2

Table e-5

1.2. Stroke and death as combined outcome
The observed risk at 15 years is **36.4%**.

No	Intervention	Absolute Risk (%) CI(95%)	Risk Ratio CI(95%)	Risk Difference CI(95%)	Total Intervened (%)
0	Natural course	38.8 (37.2, 40.5)	1 (1, 1)	0 (0, 0)	0.0
1	Maintaining SBP below 120 mmHg	39.3 (37.5, 42.3)	1.01 (0.97, 1.08)	0.5 (-1.2, 2.9)	97.8
2	Maintaining SBP below 140 mmHg	38.2 (36.5, 40)	0.99 (0.96, 1.01)	-0.5 (-1.4, 0.5)	83.5
3	Reducing SBP by 10% if above 140 mmHg	38.4 (36.9, 40.5)	0.99 (0.97, 1.02)	-0.3 (-1.4, 0.9)	82.7
4	Reducing SBP by 20% if above 140 mmHg	39.1 (37.4, 41.2)	1.01 (0.97, 1.05)	0.4 (-1.1, 2)	82.7
5	Quitting smoking	35.8 (34.2, 37.6)	0.92 (0.91, 0.94)	-2.9 (-3.6, -2.3)	25.9
6	Joint 1 + 5	36.6 (34.3, 39.2)	0.94 (0.9, 1)	-2.2 (-3.9, -0.1)	98.7
7	Joint 2 + 5	34.9 (33.8, 37)	0.9 (0.88, 0.94)	-3.8 (-4.8, -2.1)	88.6
8	Joint 3 + 5	35.5 (34, 37.4)	0.92 (0.89, 0.95)	-3.2 (-4.5, -2)	88.2
9	Joint 4 + 5	36.3 (34.4, 38.2)	0.94 (0.9, 0.97)	-2.5 (-4, -1.1)	88.2

Table e-6

1.3. Under 65 years subgroup
The observed risk at 15 years is **6.1%**.

No	Intervention	Absolute Risk (%) CI(95%)	Risk Ratio CI(95%)	Risk Difference CI(95%)	Total Intervened (%)
0	Natural course	6.3 (5, 7.9)	1 (1, 1)	0 (0, 0)	0.0
1	Maintaining SBP below 120 mmHg	5.8 (3.9, 7.9)	0.91 (0.64, 1.16)	-0.5 (-2.4, 1)	97.6
2	Maintaining SBP below 140 mmHg	5.5 (4.2, 7)	0.87 (0.72, 0.98)	-0.8 (-1.8, -0.2)	78.5
3	Reducing SBP by 10% if above 140 mmHg	5.4 (4.1, 7.2)	0.86 (0.7, 0.99)	-0.9 (-1.9, -0.1)	78.9
4	Reducing SBP by 20% if above 140 mmHg	5.5 (4.2, 7.8)	0.88 (0.67, 1.07)	-0.8 (-2.1, 0.4)	78.9
5	Quitting smoking	5.4 (4.3, 6.8)	0.85 (0.76, 0.96)	-0.9 (-1.6, -0.2)	30.9
6	Joint 1 + 5	4.8 (3.2, 6.9)	0.77 (0.53, 1.04)	-1.4 (-2.8, 0.2)	98.1
7	Joint 2 + 5	4.6 (3.4, 5.9)	0.73 (0.61, 0.88)	-1.7 (-2.7, -0.8)	86.5
8	Joint 3 + 5	4.6 (3.4, 5.9)	0.73 (0.58, 0.89)	-1.7 (-2.8, -0.7)	85.8
9	Joint 4 + 5	4.7 (3.3, 6.4)	0.75 (0.56, 0.98)	-1.6 (-2.7, -0.2)	85.8

Table e-7

1.4. Above 65 years and below 80 years subgroup
The observed risk at 15 years is **14.0%**.

No	Intervention	Absolute Risk (%) CI(95%)	Risk Ratio CI(95%)	Risk Difference CI(95%)	Total Intervened (%)
0	Natural course	14 (12.6, 15.8)	1 (1, 1)	0 (0, 0)	0.0
1	Maintaining SBP below 120 mmHg	12.1 (9.3, 15.3)	0.86 (0.69, 1.03)	-2 (-4.3, 0.4)	98.8
2	Maintaining SBP below 140 mmHg	12.6 (11.1, 14.9)	0.9 (0.81, 1)	-1.5 (-2.8, 0)	87.3
3	Reducing SBP by 10% if above 140 mmHg	12.8 (10.8, 14.9)	0.91 (0.79, 1)	-1.3 (-3.2, 0)	87.0
4	Reducing SBP by 20% if above 140 mmHg	12.4 (10.2, 15)	0.88 (0.74, 1.01)	-1.6 (-3.7, 0.1)	87.0
5	Quitting smoking	13.5 (11.7, 15.2)	0.96 (0.91, 1)	-0.6 (-1.4, 0.1)	22.1
6	Joint 1 + 5	11.6 (8.9, 14.9)	0.83 (0.64, 1.02)	-2.5 (-5.4, 0.2)	98.8
7	Joint 2 + 5	12.2 (10.4, 14.4)	0.87 (0.75, 0.97)	-1.8 (-3.5, -0.5)	91.5
8	Joint 3 + 5	12.2 (10.1, 14.3)	0.87 (0.75, 0.97)	-1.8 (-3.8, -0.5)	90.3
9	Joint 4 + 5	11.9 (9.6, 14.5)	0.85 (0.71, 1)	-2.2 (-4.3, 0)	90.3

Table e-8

1.5. Women subgroup
The observed risk at 15 years is **9.2 %**.

No	Intervention	Absolute Risk (%) CI(95%)	Risk Ratio CI(95%)	Risk Difference CI(95%)	Total Intervened (%)
0	Natural course	9.3 (8.1, 10.9)	1 (1, 1)	0 (0, 0)	0.0
1	Maintaining SBP below 120 mmHg	8.3 (6.4, 11.3)	0.9 (0.72, 1.13)	-0.9 (-2.7, 1.3)	97.2
2	Maintaining SBP below 140 mmHg	8.4 (7.4, 10.4)	0.91 (0.82, 1.03)	-0.8 (-1.8, 0.2)	83.6
3	Reducing SBP by 10% if above 140 mmHg	8.4 (7.2, 10.6)	0.9 (0.8, 1.07)	-0.9 (-2, 0.7)	82.6
4	Reducing SBP by 20% if above 140 mmHg	8.4 (6.8, 10.9)	0.9 (0.77, 1.09)	-0.9 (-2.2, 0.9)	82.6
5	Quitting smoking	8.6 (7.5, 10.4)	0.93 (0.86, 0.98)	-0.7 (-1.4, -0.2)	23.4
6	Joint 1 + 5	7.7 (6, 10.7)	0.83 (0.66, 1.04)	-1.6 (-3.3, 0.4)	98.8
7	Joint 2 + 5	7.9 (6.8, 9.9)	0.85 (0.76, 0.99)	-1.4 (-2.6, -0.1)	88.4
8	Joint 3 + 5	7.7 (6.5, 9.9)	0.83 (0.73, 1)	-1.6 (-2.8, 0)	88.0
9	Joint 4 + 5	7.7 (6.1, 10.3)	0.83 (0.71, 1.02)	-1.6 (-2.9, 0.3)	88.0

Table e-9

1.6. Men subgroup
The observed risk at 15 years is **11.6 %**.

No	Intervention	Absolute Risk (%) CI(95%)	Risk Ratio CI(95%)	Risk Difference CI(95%)	Total Intervened (%)
0	Natural course	11.6 (10.2, 13.5)	1 (1, 1)	0 (0, 0)	0.0
1	Maintaining SBP below 120 mmHg	10 (7.3, 13.2)	0.86 (0.69, 1.05)	-1.6 (-3.7, 0.5)	98.8
2	Maintaining SBP below 140 mmHg	10.2 (8.6, 12.2)	0.88 (0.77, 1)	-1.4 (-2.5, 0)	84.8
3	Reducing SBP by 10% if above 140 mmHg	10.2 (8.5, 12.5)	0.87 (0.76, 1.01)	-1.5 (-2.8, 0.1)	83.4
4	Reducing SBP by 20% if above 140 mmHg	10.1 (7.9, 12.8)	0.87 (0.71, 1.03)	-1.6 (-3.2, 0.4)	83.4
5	Quitting smoking	10.9 (9.4, 13)	0.94 (0.87, 1.02)	-0.7 (-1.7, 0.3)	30.9
6	Joint 1 + 5	9.5 (6.9, 12.5)	0.81 (0.63, 1.04)	-2.2 (-4.5, 0.5)	99.5
7	Joint 2 + 5	9.6 (8, 11.7)	0.83 (0.72, 0.96)	-2 (-3.4, -0.4)	89.7
8	Joint 3 + 5	9.5 (7.8, 12)	0.82 (0.71, 0.97)	-2.1 (-3.5, -0.4)	88.9
9	Joint 4 + 5	9.4 (7.5, 12.3)	0.81 (0.67, 1.01)	-2.2 (-4, 0.1)	88.9

Table e-10

1.7. Without hypertension medication at baseline subgroup
The observed risk at 15 years is **8.8 %**.

No	Intervention	Absolute Risk (%) CI(95%)	Risk Ratio CI(95%)	Risk Difference CI(95%)	Total Intervened (%)
0	Natural course	9.1 (8.2, 10.6)	1 (1, 1)	0 (0, 0)	0.0
1	Maintaining SBP below 120 mmHg	7.8 (6.1, 9.3)	0.85 (0.68, 1)	-1.3 (-2.7, 0)	97.8
2	Maintaining SBP below 140 mmHg	8.1 (6.8, 9.4)	0.89 (0.79, 0.96)	-1 (-1.8, -0.4)	82.1
3	Reducing SBP by 10% if above 140 mmHg	8 (6.6, 9.3)	0.87 (0.77, 0.96)	-1.1 (-2, -0.3)	81.3
4	Reducing SBP by 20% if above 140 mmHg	7.9 (6.3, 9.2)	0.86 (0.72, 0.99)	-1.2 (-2.4, -0.1)	81.3
5	Quitting smoking	8.2 (7.2, 9.6)	0.89 (0.85, 0.95)	-1 (-1.4, -0.4)	28.8
6	Joint 1 + 5	6.9 (5.1, 8.6)	0.76 (0.6, 0.93)	-2.2 (-3.4, -0.6)	98.5
7	Joint 2 + 5	7.2 (6, 8.4)	0.79 (0.71, 0.87)	-1.9 (-2.7, -1.1)	87.8
8	Joint 3 + 5	7.1 (5.7, 8.5)	0.78 (0.69, 0.88)	-2 (-2.8, -1.1)	87.9
9	Joint 4 + 5	7 (5.3, 8.4)	0.77 (0.64, 0.9)	-2.1 (-3.1, -0.9)	87.9

Table e-11

1.8. No history of heart disease at baseline subgroup
The observed risk at 15 years is **9.9%**.

No	Intervention	Absolute Risk (%) CI(95%)	Risk Ratio CI(95%)	Risk Difference CI(95%)	Total Intervened (%)
0	Natural course	9.9 (9.1, 10.9)	1 (1, 1)	0 (0, 0)	0.0
1	Maintaining SBP below 120 mmHg	8.7 (7.2, 10.9)	0.88 (0.72, 1.05)	-1.2 (-2.7, 0.6)	98.1
2	Maintaining SBP below 140 mmHg	8.9 (8.1, 10.4)	0.9 (0.84, 1)	-1 (-1.6, 0)	82.4
3	Reducing SBP by 10% if above 140 mmHg	9 (7.8, 10.4)	0.91 (0.82, 1.01)	-0.9 (-1.8, 0.2)	82.8
4	Reducing SBP by 20% if above 140 mmHg	8.9 (7.5, 10.6)	0.9 (0.77, 1.04)	-1 (-2.2, 0.4)	82.8
5	Quitting smoking	9.2 (8.3, 10.2)	0.93 (0.89, 0.99)	-0.7 (-1.1, -0.1)	26.3
6	Joint 1 + 5	8 (6.3, 10.3)	0.81 (0.66, 0.99)	-1.9 (-3.3, -0.1)	98.8
7	Joint 2 + 5	8.4 (7.3, 9.8)	0.85 (0.76, 0.94)	-1.4 (-2.3, -0.6)	88.7
8	Joint 3 + 5	8.3 (7.3, 9.8)	0.84 (0.75, 0.95)	-1.5 (-2.4, -0.5)	88.5
9	Joint 4 + 5	8.2 (6.8, 10.1)	0.83 (0.7, 0.99)	-1.6 (-2.9, -0.1)	88.5

Table e-12

2. Dementia

2.1. Death as censored.
The observed risk at 15 years is **10.8%**.

No	Intervention	Absolute Risk (%) CI(95%)	Risk Ratio CI(95%)	Risk Difference CI(95%)	Total Intervened (%)
0	Natural course	13.1 (11.7, 14.8)	1 (1, 1)	0 (0, 0)	0.0
1	Maintaining SBP below 120 mmHg	13.9 (11.7, 17.1)	1.07 (0.92, 1.26)	0.9 (-1, 3.6)	98.2
2	Maintaining SBP below 140 mmHg	13 (11.4, 15.2)	1 (0.92, 1.09)	0 (-1, 1.3)	83.0
3	Reducing SBP by 10% if above 140 mmHg	13.3 (11.5, 15.6)	1.01 (0.93, 1.12)	0.2 (-1.1, 1.6)	83.3
4	Reducing SBP by 20% if above 140 mmHg	13.7 (11.8, 16.6)	1.05 (0.92, 1.2)	0.7 (-1, 2.6)	83.3
5	Quitting smoking	12.8 (11.6, 14.5)	0.98 (0.95, 1.02)	-0.3 (-0.8, 0.2)	25.9
6	Joint 1 + 5	13.8 (11.4, 16.7)	1.05 (0.89, 1.23)	0.7 (-1.4, 3.2)	98.8
7	Joint 2 + 5	12.8 (11.1, 14.9)	0.98 (0.89, 1.09)	-0.2 (-1.3, 1.2)	88.2
8	Joint 3 + 5	13 (11.2, 15.2)	0.99 (0.9, 1.11)	-0.1 (-1.4, 1.4)	88.6
9	Joint 4 + 5	13.4 (11.4, 16.1)	1.03 (0.9, 1.16)	0.4 (-1.4, 2.2)	88.6

Table e-13

2.2. Dementia and death as combined outcome
The observed risk at 15 years is **36.7%**.

No	Intervention	Absolute Risk (%) CI(95%)	Risk Ratio CI(95%)	Risk Difference CI(95%)	Total Intervened (%)
0	Natural course	38.9 (37, 40.4)	1 (1, 1)	0 (0, 0)	0.0
1	Maintaining SBP below 120 mmHg	40.2 (38.4, 42.9)	1.03 (0.99, 1.1)	1.3 (-0.2, 3.8)	98.2
2	Maintaining SBP below 140 mmHg	38.6 (37.1, 40.7)	0.99 (0.98, 1.04)	-0.3 (-0.7, 1.6)	83.0
3	Reducing SBP by 10% if above 140 mmHg	39.5 (37.2, 41.2)	1.02 (0.99, 1.05)	0.6 (-0.6, 2)	83.3
4	Reducing SBP by 20% if above 140 mmHg	40.3 (37.8, 42.1)	1.04 (0.99, 1.07)	1.4 (-0.2, 2.8)	83.3
5	Quitting smoking	36.4 (34.3, 37.7)	0.93 (0.91, 0.94)	-2.5 (-3.6, -2.1)	25.9
6	Joint 1 + 5	37.6 (35.6, 40.1)	0.97 (0.93, 1.04)	-1.3 (-2.8, 1.4)	98.8
7	Joint 2 + 5	36.1 (34.6, 38.2)	0.93 (0.91, 0.97)	-2.8 (-3.6, -1)	88.2
8	Joint 3 + 5	36.9 (34.8, 38.2)	0.95 (0.91, 0.99)	-2 (-3.4, -0.6)	88.6
9	Joint 4 + 5	37.6 (35.2, 39.2)	0.97 (0.92, 1)	-1.3 (-3, 0.2)	88.6

Table e-14

2.3. Under 65 years subgroup
The observed risk at 15 years is **3.2%**.

No	Intervention	Absolute Risk (%) CI(95%)	Risk Ratio CI(95%)	Risk Difference CI(95%)	Total Intervened (%)
0	Natural course	4 (3.2, 5.7)	1 (1, 1)	0 (0, 0)	0.0
1	Maintaining SBP below 120 mmHg	4.3 (2.7, 6.7)	1.06 (0.75, 1.5)	0.2 (-1.1, 2.2)	97.3
2	Maintaining SBP below 140 mmHg	3.9 (3.1, 5.6)	0.97 (0.79, 1.18)	-0.1 (-0.8, 0.6)	79.2
3	Reducing SBP by 10% if above 140 mmHg	4 (2.9, 5.8)	0.99 (0.78, 1.22)	0 (-1, 0.8)	79.0
4	Reducing SBP by 20% if above 140 mmHg	4.2 (2.9, 6.2)	1.04 (0.77, 1.39)	0.2 (-1.1, 1.5)	79.0
5	Quitting smoking	4.1 (3.1, 5.8)	1.01 (0.89, 1.12)	0 (-0.4, 0.4)	30.7
6	Joint 1 + 5	4.2 (2.9, 6.5)	1.05 (0.71, 1.48)	0.2 (-1.2, 2)	98.3
7	Joint 2 + 5	4 (3, 5.8)	0.99 (0.8, 1.19)	0 (-0.9, 0.8)	86.2
8	Joint 3 + 5	4 (3.1, 5.8)	0.99 (0.81, 1.21)	0 (-0.8, 0.9)	85.5
9	Joint 4 + 5	4.2 (3, 6.1)	1.04 (0.73, 1.37)	0.2 (-1, 1.4)	85.5

Table e-15

2.4. Above 65 years and below 80 years subgroup
The observed risk at 15 years is **14.1%**.

No	Intervention	Absolute Risk (%) CI(95%)	Risk Ratio CI(95%)	Risk Difference CI(95%)	Total Intervened (%)
0	Natural course	13.8 (12.4, 15.6)	1 (1, 1)	0 (0, 0)	0.0
1	Maintaining SBP below 120 mmHg	14.4 (11.8, 17.5)	1.04 (0.85, 1.2)	0.6 (-2.2, 2.8)	98.8
2	Maintaining SBP below 140 mmHg	13.6 (12, 15.8)	0.99 (0.9, 1.08)	-0.2 (-1.3, 1.1)	87.7
3	Reducing SBP by 10% if above 140 mmHg	13.7 (11.8, 16.2)	0.99 (0.9, 1.1)	-0.1 (-1.5, 1.4)	87.1
4	Reducing SBP by 20% if above 140 mmHg	13.9 (11.7, 16.9)	1.01 (0.88, 1.14)	0.2 (-1.9, 2)	87.1
5	Quitting smoking	14 (12.7, 15.9)	1.02 (0.97, 1.06)	0.2 (-0.5, 0.9)	21.6
6	Joint 1 + 5	14.5 (11.8, 18.1)	1.05 (0.83, 1.26)	0.7 (-2.2, 3.6)	99.1
7	Joint 2 + 5	13.8 (12.1, 16.5)	1 (0.91, 1.11)	0 (-1.4, 1.6)	90.9
8	Joint 3 + 5	13.9 (12.1, 16.7)	1.01 (0.9, 1.12)	0.1 (-1.5, 1.8)	90.6
9	Joint 4 + 5	14.2 (12, 17.3)	1.03 (0.87, 1.2)	0.4 (-1.8, 2.9)	90.6

Table e-16

2.5. Women subgroup
The observed risk at 15 years is **10.7%**.

No	Intervention	Absolute Risk (%) CI(95%)	Risk Ratio CI(95%)	Risk Difference CI(95%)	Total Intervened (%)
0	Natural course	11.2 (9.7, 12.7)	1 (1, 1)	0 (0, 0)	0.0
1	Maintaining SBP below 120 mmHg	10.3 (8.3, 12.6)	0.93 (0.7, 1.1)	-0.8 (-3.6, 1.1)	98.3
2	Maintaining SBP below 140 mmHg	10.2 (8.8, 11.9)	0.91 (0.82, 1)	-1 (-2.3, 0)	84.4
3	Reducing SBP by 10% if above 140 mmHg	10.2 (8.8, 11.8)	0.91 (0.79, 1.02)	-1 (-2.6, 0.2)	82.9
4	Reducing SBP by 20% if above 140 mmHg	10.2 (8.7, 12.2)	0.92 (0.75, 1.05)	-1 (-3.1, 0.6)	82.9
5	Quitting smoking	11 (9.4, 12.7)	0.99 (0.94, 1.03)	-0.1 (-0.6, 0.3)	22.8
6	Joint 1 + 5	10.2 (8, 12.6)	0.91 (0.68, 1.08)	-1 (-3.8, 0.9)	98.5
7	Joint 2 + 5	10 (8.6, 11.8)	0.9 (0.8, 1)	-1.1 (-2.5, 0)	88.3
8	Joint 3 + 5	10 (8.7, 11.8)	0.9 (0.77, 1.01)	-1.1 (-2.8, 0.1)	88.2
9	Joint 4 + 5	10.1 (8.4, 12.3)	0.91 (0.73, 1.05)	-1.1 (-3.4, 0.5)	88.2

Table e-17

2.6. Men subgroup
The observed risk at 15 years is **6.7%**.

No	Intervention	Absolute Risk (%) CI(95%)	Risk Ratio CI(95%)	Risk Difference CI(95%)	Total Intervened (%)
0	Natural course	7 (6, 8.9)	1 (1, 1)	0 (0, 0)	0.0
1	Maintaining SBP below 120 mmHg	9.5 (7.1, 12.8)	1.36 (1.09, 1.66)	2.5 (0.6, 4.7)	98.2
2	Maintaining SBP below 140 mmHg	8.1 (6.7, 10.6)	1.15 (1.03, 1.33)	1.1 (0.2, 2.2)	83.4
3	Reducing SBP by 10% if above 140 mmHg	8.4 (6.7, 11.2)	1.2 (1.03, 1.37)	1.4 (0.2, 2.6)	83.8
4	Reducing SBP by 20% if above 140 mmHg	9.1 (6.8, 11.9)	1.29 (1.05, 1.51)	2.1 (0.3, 3.7)	83.8
5	Quitting smoking	7.5 (6.4, 9.6)	1.08 (1, 1.16)	0.6 (0, 1.1)	30.5
6	Joint 1 + 5	10.4 (7.3, 14.1)	1.48 (1.13, 1.81)	3.4 (0.9, 5.7)	98.7
7	Joint 2 + 5	8.8 (7.1, 11.2)	1.26 (1.09, 1.42)	1.8 (0.7, 3)	89.0
8	Joint 3 + 5	9.1 (7, 11.9)	1.31 (1.08, 1.49)	2.1 (0.6, 3.5)	89.7
9	Joint 4 + 5	9.8 (7.2, 13)	1.4 (1.12, 1.64)	2.8 (0.8, 4.5)	89.7

Table e-18

2.7. Without hypertension medication at baseline subgroup
The observed risk at 15 years is **8.6%**.

No	Intervention	Absolute Risk (%) CI(95%)	Risk Ratio CI(95%)	Risk Difference CI(95%)	Total Intervened (%)
0	Natural course	8.8 (8, 10.2)	1 (1, 1)	0 (0, 0)	0.0
1	Maintaining SBP below 120 mmHg	10 (7.6, 12.3)	1.14 (0.93, 1.34)	1.2 (-0.6, 3)	97.9
2	Maintaining SBP below 140 mmHg	9.1 (7.7, 10.9)	1.04 (0.95, 1.13)	0.3 (-0.5, 1.2)	81.7
3	Reducing SBP by 10% if above 140 mmHg	9.3 (7.7, 11)	1.06 (0.94, 1.16)	0.5 (-0.5, 1.4)	81.5
4	Reducing SBP by 20% if above 140 mmHg	9.8 (7.8, 11.7)	1.11 (0.94, 1.25)	1 (-0.5, 2.3)	81.5
5	Quitting smoking	8.9 (8, 10.6)	1.01 (0.97, 1.06)	0.1 (-0.3, 0.5)	28.4
6	Joint 1 + 5	10.3 (7.6, 12.6)	1.17 (0.94, 1.35)	1.5 (-0.5, 3)	98.8
7	Joint 2 + 5	9.3 (7.8, 11.1)	1.06 (0.94, 1.16)	0.5 (-0.6, 1.3)	88.1
8	Joint 3 + 5	9.5 (7.8, 11.3)	1.07 (0.94, 1.19)	0.6 (-0.6, 1.6)	88.2
9	Joint 4 + 5	9.9 (7.7, 11.8)	1.12 (0.95, 1.27)	1.1 (-0.4, 2.4)	88.2

Table e-19

2.8. Free of heart disease at baseline subgroup
The observed risk at 15 years is **8.6%**.

No	Intervention	Absolute Risk (%) CI(95%)	Risk Ratio CI(95%)	Risk Difference CI(95%)	Total Intervened (%)
0	Natural course	8.8 (8, 10)	1 (1, 1)	0 (0, 0)	0.0
1	Maintaining SBP below 120 mmHg	9.3 (7.4, 11.3)	1.06 (0.88, 1.21)	0.5 (-1, 1.8)	98.0
2	Maintaining SBP below 140 mmHg	8.9 (7.6, 10.5)	1.02 (0.91, 1.09)	0.1 (-0.8, 0.9)	83.7
3	Reducing SBP by 10% if above 140 mmHg	8.9 (7.6, 10.5)	1.01 (0.9, 1.12)	0.1 (-0.9, 1.1)	83.2
4	Reducing SBP by 20% if above 140 mmHg	9.1 (7.6, 11)	1.04 (0.89, 1.16)	0.3 (-0.9, 1.4)	83.2
5	Quitting smoking	8.8 (7.8, 10)	1 (0.96, 1.04)	0 (-0.4, 0.4)	26.3
6	Joint 1 + 5	9.4 (7.6, 11.3)	1.07 (0.89, 1.23)	0.6 (-1, 1.9)	98.9
7	Joint 2 + 5	9 (7.7, 10.4)	1.02 (0.9, 1.11)	0.2 (-0.9, 1)	88.7
8	Joint 3 + 5	9 (7.6, 10.7)	1.02 (0.91, 1.13)	0.2 (-0.8, 1.2)	88.8
9	Joint 4 + 5	9.2 (7.6, 11.1)	1.05 (0.9, 1.16)	0.4 (-0.9, 1.6)	88.8

STROBE Statement

Checklist of items that should be included in reports of cohort studies

	Item No	Recommendation	Page No
Title and abstract	1	(a) Indicate the study's design with a commonly used term in the title or the abstract	3
		(b) Provide in the abstract an informative and balanced summary of what was done and what was found	3
Introduction			
Background/rationale	2	Explain the scientific background and rationale for the investigation being reported	4
Objectives	3	State specific objectives, including any prespecified hypotheses	5
Methods			
Study design	4	Present key elements of study design early in the paper	5 – 7
Setting	5	Describe the setting, locations, and relevant dates, including periods of recruitment, exposure, follow-up, and data collection	5 – 7
Participants	6	(a) Give the eligibility criteria, and the sources and methods of selection of participants. Describe methods of follow-up	5 – 7
		(b) For matched studies, give matching criteria and number of exposed and unexposed	-
Variables	7	Clearly define all outcomes, exposures, predictors, potential confounders, and effect modifiers. Give diagnostic criteria, if applicable	5 – 8
Data sources/ measurement	8*	For each variable of interest, give sources of data and details of methods of assessment (measurement). Describe comparability of assessment methods if there is more than one group	7 OR*:3
Bias	9	Describe any efforts to address potential sources of bias	9
Study size	10	Explain how the study size was arrived at	Figure 1
Quantitative variables	11	Explain how quantitative variables were handled in the analyses. If applicable, describe which groupings were chosen and why	OR: 1
Statistical methods	12	(a) Describe all statistical methods, including those used to control for confounding	7 – 9
		(b) Describe any methods used to examine subgroups and interactions	7 – 9
		(c) Explain how missing data were addressed	OR: 4
		(d) If applicable, explain how loss to follow-up was addressed	7 – 9
		(e) Describe any sensitivity analyses	9
Results			
Participants	13*	(a) Report numbers of individuals at each stage of study—eg numbers potentially eligible, examined for eligibility, confirmed eligible, included in the study, completing follow-up, and analysed	Figure 1
		(b) Give reasons for non-participation at each stage	Figure 1

		(c) Consider use of a flow diagram	Figure 1
Descriptive data	14*	(a) Give characteristics of study participants (eg demographic, clinical, social) and information on exposures and potential confounders	Table 1
		(b) Indicate number of participants with missing data for each variable of interest	Table 1
		(c) Summarise follow-up time (eg, average and total amount)	9
Outcome data	15*	Report numbers of outcome events or summary measures over time	9, 10
*OR: Online resources			
