# Supplementary material for: Hypothetical blood-pressure-lowering interventions and risk of stroke and dementia
Source: Eur J Epidemiol. 2020 Nov 27;36(1):69–79. doi: 10.1007/s10654-020-00694-5 (PMC7847439; doi:10.1007/s10654-020-00694-5)
Supplement: Supplementary file 2 — Supplementary file2 (PDF 322 kb) [file 10654_2020_694_MOESM2_ESM.pdf]

Versión en español realizada por Dr. Rojas-Saunero, no revisada por la editorial.

Spanish version prepared by Dr. Rojas-Saunero and not reviewed by the publisher.

Intervenciones hipotéticas para disminuir la presión arterial, y el riesgo de accidente cerebrovascular y demencia

L. Paloma Rojas-Saunero, MD[1*]; Saima Hilal PhD[1,2,3*]; Eleanor J. Murray Sc.D [4,5], Roger W. Logan PhD [4]; M. Arfan Ikram PhD [1]; Sonja A. Swanson, Sc.D[1,4]

Afiliaciones:

1. Department of Epidemiology, Erasmus University Medical Center, Rotterdam, the Netherlands

2. Department of Radiology and Nuclear Medicine, Erasmus University Medical Center, Rotterdam, the Netherlands

3. Saw Swee Hock School of Public Health, National University of Singapore and National University Health System, Singapore

4. Department of Epidemiology, Harvard TH Chan School of Public Health, Boston, MA, USA

5. Department of Epidemiology, Boston University School of Public Health, Boston, MA, USA

* *These authors contributed equally to the manuscript*

Autor correspondiente:

L. Paloma Rojas-Saunero MD, Department of Epidemiology, Erasmus University Medical Center.

PO Box 2040, 3000 CA Rotterdam, the Netherlands. Tel: +31107044294. E-mail address:

l.rojassaunero@erasmusmc.nl

Agradecimientos:

Agradecemos a todos los participantes y al personal del Estudio de Rotterdam por su tiempo y compromiso con el estudio.

RESUMEN

Objetivo: Estimar los efectos de intervenciones hipotéticas en la presión arterial sistólica (PAS) y en el hábito de fumar en el riesgo de accidente cerebrovascular (ACV) y el riesgo de demencia usando datos de 15 años de seguimiento dentro del Estudio de Rotterdam.

Métodos: Usamos datos de 4930 personas, con edad de 55-80 años, sin antecedente de ACV, deterioro cognitivo, demencia y seguidos durante 15 años dentro del Estudio de Rotterdam, una cohorte poblacional. Definimos las siguientes intervenciones sostenidas en la PAS (1) mantener la PAS debajo de 120 mmHg, (2) mantener la PAS debajo de 140 mmHg, (3) reducir la PAS en un 10% si es mayor a 140 mmHg, (4) reducir la PAS en 20% si es mayor a 140 mmHg, e intervenciones que combinan la intervención de dejar de fumar con cada una de las estrategias propuestas para disminuir la PAS. Consideramos como evento el caso incidente de ACV y el diagnóstico incidente de demencia. Aplicamos la g-formula paramétrica para ajustar por variables confundidoras fijas y que varían en el tiempo.

Resultados: El riesgo observado de ACV a 15 años fue del 10.7%. Comparando con la intervención no especificada ("el curso natural"), todas las intervenciones que reducen la PAS estuvieron asociadas con una reducción del riesgo de ACV de alrededor del 10% (ej. Reducir la PAS en 20% si es mayor a 140mmHg, riesgo relativo: 0.89; IC95%: 0.76, 1). Intervenciones conjuntas en la PAS y dejar de fumar disminuyeron más el riesgo de ACV (ej. riesgo relativo: 0.83; IC95%: 0.71, 0.94). Ninguna de las intervenciones especificadas estuvieron asociadas con un cambio substancial en el riesgo de demencia.

Conclusiones: Nuestro estudio sugiere que intervenciones conjuntas en la PAS y dejar de fumar pueden disminuir el riesgo de ACV en la edad tardía, mientras que el potencial para reducir el riesgo de demencia no fue observado.

Palabras clave: hipertensión arterial, demencia, ACV, ensayo ideal, g-formula

INTRODUCCIÓN

El aumento de la esperanza de vida en las últimas décadas tiene implicaciones profundas en la ocurrencia de varias enfermedades. Como resultado del rápido envejecimiento demográfico, se espera que incremente rápidamente la carga de las enfermedades relacionadas con la edad, tales como el accidente cerebro-vascular (ACV) y la demencia [1]. Como tal, se necesitan estrategias efectivas para prevenir o retrasar el desarrollo de estas enfermedades. Dirigirse a personas sanas en relación a enfermedades crónicas relacionadas con la edad tiene el potencial de tener el mayor impacto en la salud de la población en general[2].

La hipertensión arterial es un factor de riesgo modificable reconocido para el ACV[3] y asimismo ha sido propuesto como un factor de riesgo para demencia[4], a pesar de que los mecanismos específicos biológicos son heterogéneos y menos claros, tales como el efecto del daño vascular crónico encubierto (isquemia, microhemorragia o atrofia)[5]. Los ensayos clínicos aleatorizados han reportado que el tratamiento de la hipertensión arterial reduce el riesgo del primer evento de ACV en un 35-40% entre los pacientes ancianos con hipertensión sistólica[6,7]. Algunos estudios observacionales han evaluado la asociación entre los factores relacionados al estilo de vida (ej. dieta no saludable, fumar, beber bebidas alcohólicas y la inactividad física) y el riesgo de ACV, estos estudios reportan que entre un 35 y 55% de los eventos de ACV son atribuidos a los factores relacionados al estilo de vida[8-10]. A diferencia del ACV, la evidencia de los ensayos clínicos y estudios observacionales que respaldan los efectos de la disminución de la presión arterial en el riesgo de demencia son limitados. En general, tales ensayos no fueron diseñados considerando la demencia como el evento primario, se enfocaron en grupos altamente seleccionados de pacientes y tuvieron un periodo de seguimiento corto (por ej. dos a cuatro años de seguimiento)[6, 11-18]. En contraste, los estudios observacionales previos evaluaron los efectos de la presión arterial sistólica o el tratamiento antihipertensivo conducidos en cohortes poblacionales con un seguimiento más prolongado[19,20]. Sin embargo, en ambos escenarios, la mayoría de los estudios no han sido conducidos para estimar el efecto de una estrategia de tratamiento sostenida con la consideración adecuada de las variables confundidoras que varían en el tiempo y con atención al evento competitivo de la muerte.

Ya que un ensayo clínico de largo seguimiento en una población general no ha sido conducido (y es poco factible), tomar decisiones hoy relacionadas a intervenciones dinámicas en la presión arterial y en otros cambios de hábitos de vida (ej. dejar de fumar) pueden ser empíricamente informadas utilizando datos observacionales para emular el "ensayo ideal" (*En inglés: target trial emulation*) [21–25]. La emulación del ensayo ideal requiere

una clara especificación de los elementos del protocolo del ensayo clínico, y cuando se evalúan intervenciones sostenidas en el tiempo, los métodos analíticos conocidos como “g-methods” son requeridos para contemplar apropiadamente el sesgo de confusión que depende del tiempo [26]. Estudios previos han demostrado como los resultados de estudios observacionales pueden aproximarse a los resultados de ensayos clínicos aleatorizados cuando el marco del “ensayo ideal” es implementado [27, 28]. En este estudio, emulamos un ensayo ideal para estimar el efecto sostenido de varias intervenciones hipotéticas en el control de la presión arterial sistólica (PAS), incluyendo combinaciones con una intervención en dejar de fumar durante el seguimiento, en el riesgo del primer ACV y en el desarrollo de demencia, utilizando datos del seguimiento de 15 años en el Estudio de Rotterdam.

METODOS

Iniciamos describiendo brevemente las especificaciones del ensayo ideal, y luego continuamos describiendo cómo emulamos el ensayo utilizando datos del Estudio de Rotterdam. Una comparación detallada del ensayo ideal y la emulación utilizando datos observacionales se encuentra en materiales en línea (online resource, Table e-1)

Especificaciones del ensayo ideal

Los elementos clave del ensayo ideal incluyen:

Criterios de elegibilidad: Personas de 55-80 años de edad, sin historia previa de ACV, accidente isquémico transitorio, enfermedad de Parkinson, Parkinsonismo, deterioro cognitivo o diagnóstico de demencia.

Estrategias de tratamiento: Las personas elegibles son asignadas a una de las siguientes estrategias que serán sostenidas durante el estudio: (1) mantener la PAS debajo de 120 mmHg, (2) mantener la SBP debajo de 140 mmHg, (3) reducir la PAS en 10% si es mayor a 140 mmHg, (4) reducir la SBP en 20% si es mayor a 140 mmHg. La forma en la que se seguirán estas estrategias no está pre-definida (ej. la PAS puede reducirse modificando hábitos de vida o con medicación); volvemos a este punto en la discusión. Dado que los efectos de fumar en la salud son conocidos, consideramos la intervención de (5) dejar de fumar, y también cuatro intervenciones combinando (5) con (1), (2), (3), y (4). Comparamos todas estas estrategias con el “curso natural”, que representa la estrategia no preespecificada. Cabe recalcar que las estrategias (1) y (2) se alinean con estrategias recientemente estudiadas en un ensayo aleatorizado [17], mientras que (3) y (4) son estrategias que son probablemente más alcanzables en la práctica.

Registro del evento: Los dos eventos principales de interés fueron el primer evento de ACV y el diagnóstico de demencia durante 15 años de seguimiento, registrados por un vínculo continuo entre los registros clínicos electrónicos y las evaluaciones cognitivas periódicas. Dado que la muerte es un evento competitivo, también consideramos el evento combinado con muerte como un análisis secundario.

Inicio y fin del seguimiento: Cada persona eligible fue seguida desde el momento que cumplieron con los criterios de elegibilidad descritos anteriormente. Fueron seguidas hasta el desarrollo del primer evento de ACV, diagnóstico de demencia, muerte, última fecha de seguimiento o la fecha administrativa que corresponde a 15 años desde la fecha de inicio.

Emulación del ensayo ideal

Diseño de estudio y participación pública: Para emular el ensayo ideal descrito, utilizamos datos del Estudio de Roterdam (The Rotterdam Study (RS)), un estudio de cohorte prospectiva poblacional que incluye personas de edad media y personas mayores, viviendo en el distrito de Ommoord, en la ciudad de Rotterdam, Países Bajos. Las personas que vivían en el distrito fueron invitadas a participar en la cohorte entre 1990 y 1993. Todas las personas participaron contestando cuestionarios, recibieron examinaciones clínicas y físicas y se recolectó muestras de sangre al inicio (1990-1993) y durante las visitas de seguimiento en 1993-1995, 1997-1999, 2002-2005 [29].

Criterios de elegibilidad: Los mismos especificados anteriormente. El antecedente de ACV, accidente isquémico transitorio, enfermedad de Parkinson, Parkinsonismo y demencia fueron realizados en evaluaciones en domicilio y revisando los registros médicos, se definió deterioro cognitivo a un valor debajo de 26 en el test Mini Mental State Examination (MMSE) durante la primera visita. De esta forma, de 7983 personas que participaron al inicio, 5193 fueron consideradas elegibles para el estudio en base a los criterios descritos anteriormente. Adicionalmente requerimos información completa en la PAS, índice de masa corporal (BMI), hábito de fumar y/o medicación antihipertensiva, dando un tamaño de muestra final de 4930 participantes (Figura 1). Las personas participantes con datos perdidos, que representan un 5% de la población elegible, fueron, en promedio, tres años mayores que quienes fueron incluidas, tuvieron en mayor frecuencia una educación primaria y tuvieron una mayor prevalencia de enfermedad cardiaca y diabetes al inicio del estudio (online resource, Table e-2).

Estrategias de tratamiento: Las mismas especificadas anteriormente. La PAS se midió dos veces utilizando un esfigmomanómetro de cero aleatorio, en posición sentado, y la media de ambas mediciones fueron calculadas

para cada visita de seguimiento. El hábito de fumar cigarrillos fue recolectado utilizando un cuestionario detallado y fue categorizada como nunca fumó, fuma actualmente, fumó previamente.

Registro del evento: El evento incidente de ACV fue recolectado mediante un monitoreo continuo entre los datos recolectados propios al estudio y el vínculo con los registros clínicos digitalizados de médicos de cabecera y la información del Instituto Regional de Cuidados de Salud Mental para Pacientes Ambulatorios. Para las personas participantes que se mudaron fuera del distrito o que fueron a vivir a residencias para personas mayores se revisó de forma regular las historias clínicas y el contacto con sus médicos tratantes. Investigadores clínicos revisaron todos los casos potenciales de ACV utilizando la documentación del alta hospitalaria e información de médicos de cabecera y médicos que atienden en las residencias para personas mayores. Un equipo de especialistas en neurología vascular verificaron el diagnóstico de ACV [30,31]. En concordancia con los criterios definidos por la Organización Mundial de la Salud, ACV fue definido como síndrome clínico de desarrollo rápido debido a una perturbación focal o global de la función cerebral de origen vascular y de más de 24 horas de duración.

El diagnóstico de demencia fue recolectado mediante un tamizaje durante las visitas definidas en la cohorte, utilizando el test MMSE y el Geriatric Mental Schedule (GMS) de nivel orgánico. Quienes tuvieran un valor positivo (MMSE<26 o GMS nivel orgánico >0) recibieron la evaluación basada en el Cambridge Examination for Mental Disorders in the Elderly. Un panel de consenso liderado por un o una especialista en neurología estableció el diagnóstico final de acuerdo a los criterios estándares de demencia (DSM-III-R). Adicionalmente, las personas fueron seguidas continuamente para evaluar el diagnostico de demencia en base al vínculo con los registros clínicos de médicos de cabecera, como fue para el detectar los casos de ACV [32,33].

El estado vital fue obtenido en base al registro poblacional semanal municipal y mediante información de las bases de datos de registros clínicos hospitalarios y de atención primaria. La mortalidad por todas las causas se definió cuando una persona falleció por cualquier causa durante el seguimiento del estudio.

Inicio y fin de seguimiento: Definimos el inicio del estudio como la fecha de inclusión al Estudio de Roterdam para las personas que cumplieran con los criterios de inclusión en esta fecha. Las personas participantes del estudio fueron seguidas desde el inicio hasta el desarrollo de ACV, diagnóstico de demencia, muerte, censura por pérdida de seguimiento o hasta 15 años después de la fecha de inclusión, lo que ocurra primero. Definimos pérdida de seguimiento al evento de ACV a: participantes que faltaron a una de las visitas o fueron perdidos durante el seguimiento fueron censurados utilizando la fecha correspondiente al último año en el cual podían haber atendido a la visita que les correspondía. De las personas incluidas que no desarrollaron el evento o que

murieron durante el seguimiento 283 (9%) fueron perdidas durante la primera visita, 408 (13%) después de la segunda visita, 230 (7%) después de la tercera visita, y 2285 (71%) fueron censuradas posterior a la cuarta visita. Para el análisis con el evento de demencia, las personas participantes se siguieron hasta el diagnóstico de demencia, muerte o la censura previamente definida. De las 4930 personas incluidas, 280 (9%) fueron perdidas después de la primera visita, 398 (12%) después de la segunda visita, 194 (6%) después de la tercera visita, y 2324 (73%) fueron censuradas posterior a la cuarta visita (Figura 1).

Análisis estadístico:

Para estimar el riesgo de ACV y demencia bajo las intervenciones hipotéticas descritas, utilizamos la g-formula paramétrica, una extensión de estandarización para exposiciones y variables confundidoras que cambian en el tiempo. Asumiendo que no existen variables confundidoras no medidas y que los modelos son correctos, este método provee un estimado del riesgo del evento bajo una adherencia completa a las diferentes intervenciones sostenidas hipotéticas [22,25,34–36].

Los pasos simplificados de la g-formula paramétrica, utilizando ACV como el evento de desenlace, se describen a continuación:

1. Realiza un modelo de regresión paramétrico para cada una de las variables que cambian en el tiempo, como una función de las variables basales y la historia de las covariables para las personas con seguimiento hasta el tiempo *k*.
2. Realiza un modelo de regresión paramétrico para ACV y muerte, como una función de las variables basales y la historia de covariables para las personas que fueron seguidas hasta el tiempo *k*, utilizando un modelo de regresión logística agrupado (*en inglés: pooled logistic regression*) para aproximarnos al riesgo del tiempo-al-evento.
3. Utiliza una simulación de Monte Carlo para recrear la historia de la información para una pseudopoblación de 10000 personas simuladas.
    a. Las variables basales se seleccionan aleatoriamente con reemplazo de la población original.
    b. Los valores de las variables que cambian en el tiempo se obtienen de la distribución paramétrica realizada en Paso 1.
    c. El valor de las covariables que serán “intervenidas” se basa en la estrategia definida (saltar este paso para la “estrategia del curso natural”).

d. El riesgo, en base a la a probabilidad predicha de demencia y muerte, es calculado para cada persona en la pseudopoblación.

4. Calcula el promedio del riesgo predicho de ACV y muerte a 15 años en la pseudopoblación.
5. Calcula la diferencia del riesgo entre cada estrategia y el curso natural.
6. Repite los pasos previos en 500 muestras utilizando el método de bootstrap para estimar el intervalo de confianza al 95% (IC95%).
7. Para cada estrategia repita el paso 3 a 6.

Los mismos pasos se realizaron utilizando demencia como evento. El análisis principal consistió en modelos con confundidores basales (variables fijas) incluyendo: edad como polinomio cuadrático, sexo, portación de APOE-ε4, historia de diabetes tipo 2, historia de enfermedad cardiaca, nivel de educación, PAS basal como polinomio cúbico. Adicionalmente, incluimos covariables que varían en el tiempo; el proceso de las visitas, PAS, colesterol, índice de masa corporal (BMI), consumo de alcohol, hábito de fumar, medicación antihipertensiva y la incidencia de las siguientes patologías: enfermedad cardiaca, diabetes, cáncer, accidente isquémico transitorio, enfermedad de Parkinson, Parkinsonismo. Los detalles de las mediciones se encuentran disponibles en los materiales suplementarios en línea. Cuando ACV es el evento principal, el diagnóstico de demencia fue incluido como una covariable que varía en el tiempo, y viceversa. Todas las variables que fueron medidas durante el proceso de la visita fueron modeladas bajo la condición de haber atendido a la visita primariamente (Online resource, Table e-3). Para evaluar las especificaciones del modelo, estimamos la diferencia entre el promedio observado y el promedio predicho de cada variable (online resource, Figure e-1, Figure-2). Asimismo conducimos un análisis de sensibilidad reorganizando el orden de las variables que varían en el tiempo, para evaluar las especificaciones del modelo.

Los resultados se presentan como el efecto causal promedio bajo cada una de las intervenciones hipotéticas a 15 años de seguimiento, comparado con el curso natural en términos de riesgo relativo y diferencia de riesgos. Para cada intervención reportamos la proporción acumulada de participantes en quienes hubiésemos tenido que intervenir durante el seguimiento para que adhieran a la estrategia. Adicionalmente presentamos las curvas de incidencia acumulada, comparando el riesgo bajo el curso natural y una estrategia de tratamiento conjunta.

Análisis de riesgos competitivos: Durante el seguimiento, las y los participantes pueden morir antes de desarrollar ACV y demencia, y las intervenciones pueden afectar el riesgo de muerte. Por esta razón, estimamos el riesgo de ACV y demencia considerando que las personas pueden morir. Esto significa que el efecto en el evento principal es a través de todas las vías entre la intervención y el evento, incluyendo aquellas vías mediadas

por el evento competitivo. Adicionalmente realizamos el análisis primario considerando muerte como un evento de censura, sin embargo la interpretación en este escenario emula a un mundo contrafactual en el cual la muerte podría ser completamente prevista, lo cual no es realista y se basa en un supuesto más fuertes en relación a tener todas las variables confundidoras medidas [37]. Finalmente, realizamos un análisis considerando el efecto de cada intervención en el evento compuesto con muerte (ej. ACV y muerte, demencia y muerte).

Análisis de subgrupos: Repetimos el análisis primario para los siguientes subgrupos: edad entre 55 y 65 años; edad entre 66 y 80 años; mujeres y hombres; sin medicación hipertensiva al inicio del estudio; y libre de enfermedad cardíaca al inicio del estudio.

Todos los análisis utilizando la g-formula fueron realizados con el programa SAS 9.4 y la macro GFORMULA que se encuentra públicamente disponible en http://www.hsph.harvard.edu/causal/software. El código de SAS para la macro GFORMULA utilizado para nuestro análisis principal se encuentran disponibles en el siguiente repositorio https://github.com/palolili23/ht_trial_gformula.

RESULTADOS

La Tabla 1 muestra las características basales de las personas participantes. La edad promedio de los y las participantes fue de 66 años y 57% fueron mujeres. La media de PAS al inicio del estudio fue 137 mmHg, y 24% eran fumadores al momento.

Riesgo de ACV: Durante 15 años de seguimiento, hubieron 490 casos incidentes de ACV y 1234 muertes. El riesgo observado a 15 años de ACV fue 10.3% y bajo el curso natural simulado fue de 10.3% (IC95%: 9.3, 11.5). El riesgo de ACV bajo las diferentes estrategias de tratamiento hipotéticas se presentan en la Tabla 2. En general, todas las intervenciones que disminuyen la PAS debajo de un umbral reducieron el riesgo de ACV en aproximadamente un 10% comparado con el curso natural, durante el periodo de estudio. Si bien todas las intervenciones en la PAS tuvieron una asociación similar con el riesgo de ACV, la estrategia de tratamiento más intensiva ("mantener la PAS debajo de 120 mmHg") requirió intervenir en un 98% de la población en algún momento durante el seguimiento, lo cual incluye un 15% más de personas, comparado con las otras estrategias.

En cambio, dejar de fumar se asoció con una reducción en el riesgo de ACV en 7% (RR IC95%: 0.89, 0.97) comparado con el curso natural, y requirió intervenir en 26% de la población. Todas las intervenciones combinadas mostraron una reducción mayor en el riesgo de ACV. Por ejemplo, disminuir la PAS en 20% si es

mayor a 140 mmHg y dejar de fumar se asoció con un 17% (RR IC95% : 0.71, 0.94) en la reducción del riesgo de ACV durante el periodo de estudio comparado con el curso natural, como se observa en la Figura 2A.

Riesgo de demencia: Durante los 15 años de seguimiento, hubieron 431 casos de demencia y 1303 muertes. El riesgo de demencia observado a 15 años fue de 8.9% y bajo el curso natural simulado fue de 9.2% (IC95%: 8.2, 10.3%). El riesgo de demencia bajo las diferentes intervenciones hipotéticas se presentan en la Tabla 3. En general, ninguna de las estrategias de tratamiento enfocadas en la PAS se asociaron con cambios substanciales en el riesgo de demencia. Por ejemplo, la estrategia "disminuir la PAS debajo de 120 mmHg" se asoció a un incremento en 6% (RR 95% CI: 0.90, 1.24) en el riesgo de demencia comparada al curso natural. Este patrón se observó también para la estrategia de dejar de fumar y en las estrategias que combinan la disminución de la PAS y dejar de fumar. Por ejemplo, disminuir la PAS en 20% si es mayor a 140 mmHg y dejar de fumar se asoció a un incremento en el riesgo de demencia del 5% (RR 95% CI: 0.92, 1.20) como se observó en la Figure 3A.

Análisis alternativos para el evento competitivo: Dado que el evento de muerte fue modelado como un evento competitivo para ambos eventos (ACV y demencia), presentamos el efecto de la intervención "disminuir en 20% la PAS si es mayor a 140 mmHg y dejar de fumar" en el riesgo de muerte en la figura 2B y figura 3b respectivamente. Tratar la muerte como un evento de censura y como parte de un evento combinado no modificaron nuestros resultados significativamente, cómo se presenta en los materiales en línea (online resource, Tabla e-4, e-5, e-11, e-12).

Análisis de subgrupo: La tabla 4 provee las estimaciones para la estrategia de tratamiento "disminuir en 20% la PAS si es mayor a 140 mmHg y dejar de fumar" en el riesgo de ACV y demencia en diferentes subgrupos (edad, sexo, sin medicación antihipertensiva al inicio del estudio, libre de enfermedad cardíaca al inicio del estudio) comparada con el curso natural. Las estimaciones fueron relativamente consistentes para ACV, con excepción de las personas menores a 65 años dónde se observe una asociación más fuerte (RR: 0.75, CI95%: 0.56, 0.98). Para el riesgo de demencia, el análisis de subgrupos presentó resultados similares. Las estrategias de tratamiento adicionales se presentan en el material en línea, Table e-6 a e- 11, e-14 a e-19.

DISCUSIÓN

Nuestro estudio sugiere que intervenir en la presión arterial podría reducir el riesgo de ACV en aproximadamente un 10% durante15 años de seguimiento, en un escenario poblacional, y a la vez, combinar estas intervenciones con una intervención en dejar de fumar podría resultar en una reducción del 18%. Al

contrario, nuestro estudio es consistente al evidenciar que las mismas intervenciones tienen un efecto nulo u opuesto en el riesgo de demencia, tomando en cuenta que estas estimaciones podrían estar afectadas por cómo las intervenciones podrían reducir el riesgo de muerte.

Nuestros resultados en el riesgo de ACV son compatibles en dirección pero no en magnitud de los efectos estimados en estudios previos. Un metaanálisis previo de ensayos clínicos aleatorizados mostró que una reducción de 10 mmHg en la presión arterial reduce el riesgo de ACV en un 27%, si bien este efecto se observó en una población de personas de alto riesgo con enfermedad cardiovascular [38]. El estudio observacional más comparable al nuestro fue un estudio que emula estrategias de tratamiento hipotéticas para reducir la PAS en personas sanas en Noruega con edad media (promedio de edad basal de 46.1 años o 20 años más jóvenes que en nuestro estudio), utilizando la g-formula. Este estudio mostró que, en promedio, una reducción de 23 mmHg en personas con PAS de 120 mmHg o mayor resulta en una reducción de un 45% en el riesgo de ACV a los 15 años de seguimiento[35]. La diferencia en el riesgo estimado puede deberse a la diferencia en el promedio etáreo de la población de estudio. Asimismo, no hubieron diferencias en la reducción de riesgo proporcional reportada en ensayos que involucraron personas con presión arterial <130 mmHg y en quienes están en alto riesgo (≥160 mmHg) [38]. Nuestro estudio también contribuye a los estudios previos al considerar una intervención conjunta con dejar de fumar, si bien nuestras estrategias combinadas reducen el riesgo de ACV en un menor medida (18%) que la reportada en estudios observacionales previos (35-55%) [8–10].

Para comparar los resultado de las intervenciones hipotéticas en la PAS (disminuir debajo de 120 o 140 mmHg en el tiempo) en el riesgo de demencia con la literatura previa, debemos considerar las diferencias en los criterios de elegibilidad, estrategias de tratamiento y en las decisiones analíticas. Nuestro ensayo ideal se asimila en diseño a las estrategias de tratamiento como en el ensayo Systolic Blood Pressure Intervention Trial (SPRINT) MIND, sin embargo este ensayo consideró como personas elegibles a quienes tuvieran un riesgo de enfermedad cardiovascular [17], lo cual representa un pequeño subgrupo en nuestra cohorte poblacional (específicamente, cerca de 290 personas en el estudio de Rotterdam cuentan con los mismos criterios al inicio del estudio, basados en las mediciones de la PAS, presencia de enfermedad cardiovascular diferente a ACV e historia de diabetes). Similarmente, ensayos clínicos previos han sido primariamente diseñados para evaluar el efecto de medicación antihipertensiva en el riesgo de ACV y no considerando el riesgo de demencia como evento principal, asimismo fueron diseñados para un subgrupo específico de personas que requerían tratamiento. Los criterios de elegibilidad en otros ensayos clínicos incluyeron tener historia de ACV, ser mayor a 80 años de edad y tener una PAS mayor a 160 mmHg [6,11–18]. Más aún, evaluar la comparación de nuestros hallazgos con estudios

observacionales previos requiere cuidado. Un reciente metaanálisis por Ding et al. ha estudiado el efecto de tomar una medicación antihipertensiva o una mediación específica y el riesgo de demencia, estratificado por PAS, incluyendo cinco cohortes poblacionales [19]. Sin embargo, evaluaron el efecto de estar en tratamiento al inicio del estudio y solo incluyeron variables confundidoras basales en el ajuste. Asimismo, Want et al. estratificaron a los individuos en base a los patrones longitudinales de la PAS (normotensión/hipertensión en la edad media y edad tardía), pero las covariables sólo estuvieron medidas en dos de las seis visitas [39]. Al contrario, en este estudio evaluamos el efecto sostenido de disminuir la PAS durante el periodo de seguimiento, considerando covariables que se actualizan en el tiempo. Por último, consideramos que nuestros hallazgos reflejan la relevancia de considerar al evento competitivo de la muerte por otras causas, y cómo las estimaciones pueden estar afectadas por el efecto de las intervenciones en el riesgo de muerte, por lo cual dan una mirada más comprensiva de las implicaciones de los resultados. Esto será especialmente importante cuando estratificamos por características que tienen una distribución de sobrevida diferente, como observamos en la diferente dirección de los efectos al comparar mujeres vs. hombres [40]. Reconsiderar estos puntos como parte de cómo enmarcamos la pregunta de investigación y las decisiones analíticas utilizando datos observacionales, tendrán un impacto directo en la interpretación den nuestros resultados y en la traducción clínica.

Considerando una base de datos rica en información observacional a nivel poblacional, de alta calidad y de mediciones frecuentes de los eventos y variables clave, y el uso de la g-formula paramétrica para contemplar la estructura compleja del sesgo de confusión considerado, emulamos un "ensayo ideal" que puede ser de interés en salud pública pero no que sería fácilmente conducido como un ensayo clínico. Sin embargo, como todo análisis de datos observacionales, varios supuestos deben ser evaluados. La posibilidad de tener variables confundidoras que no fueron contempladas, y en particular no pudimos ajustar por covariables como ser el tipo de medicación antihipertensiva, LDL (separada del colesterol total), glucosa y fragilidad en nuestro análisis. Asimismo, utilizamos la prueba de MMSE como herramienta de tamizaje y excluimos personas con enfermedad de Parkinson o síntomas de Parkinsonismo al inicio del estudio, pero es posible que otras personas tuvieran deterioro cognitivo subclínico y que hayan sido incluidas en nuestro análisis basal y durante el seguimiento[41]. El hábito de fumar también es vulnerable a error de medición, si bien evaluamos la consistencia de nuestras mediciones en el tiempo. Además, la g-formula paramétrica se basa en varios supuestos de modelado. Como reportamos en material suplementario (online resource, Figure e-1, Figure e-2), observamos un acuerdo entre los valores estimados promedio de cada variable (evento y covariables) bajo el curso natural con los valores observados, lo cual apoya pero no prueba que las especificaciones de los modelos sean correctas. Más aún, no

evaluamos los efectos de las intervenciones hipotéticas en fenotipos clínicos específicos de cada enfermedad, ya que el número de participantes para cada subtipo clínico desagregados es pequeño, además complejiza el análisis ya que cada subtipo sería considerado como un evento competitivo para el otro. Sin embargo, el efecto de disminuir la presión arterial sistólica afectaría el riesgo de cada subtipo clínico en una magnitud diferente y debería ser dirigido en estudios futuros.

Finalmente, otro punto clave para reflexionar al interpretar nuestras estrategias de tratamiento especificadas es que no especificamos, en efecto, cómo se reduciría la PAS. Esto significa que nuestros estimados están basados en el supuesto de consistencia para el cual disminuir la PAS mediante diferentes formas (ej. cambios dietarios, medicación, cambios en el estilo de vida), tendrían el mismo efecto en el riesgo de ACV o demencia, de otra manera, nuestros estimados se interpretan como un efecto ponderado de diferentes estrategias para disminuir la PAS, con pesos determinados por la frecuencia en la que cada estrategia particular se presenta en nuestra población específica [42,43]. Estudios futuros que tengan información más detallada de los tratamientos que reducen la PAS se necesitan para desenredar la relevancia de la variación en el tratamiento y construir en base a este estudio inicial. Más aún, dejar de fumar es una de muchas intervenciones conductuales o relacionadas al estilo de vida que podrían ser exploradas, como también otros factores metabólicos descritos en las guías actuales [3,4]. Implementar el marco del “ensayo ideal” y definir la pregunta de investigación para estudiar estrategias de tratamiento más refinadas son el siguiente paso crucial. Realizar esto requiere datos longitudinales, ricos en información específica de las intervenciones a ser estudiadas; el nivel de especificidad de la pregunta de investigación que pueda ser estudiada estará limitada por la disponibilidad de los datos. Por este motivo, si bien hay limitaciones claras en relación a la ambigüedad de las intervenciones, representan una mejora (en términos de claridad y para informar la toma de decisiones) con respecto a los estudios etiológicos que abordan los efectos de la PAS con una versión simplificada de la complejidad de los datos reales, y un paso hacia los tipos de intervenciones que podemos considerar en la práctica.

Dadas las consideraciones anteriores, estudiar las intervenciones a nivel poblacional como se hace en este estudio es particularmente adecuado para la investigación en salud pública, en el sentido de que podemos comprender mejor cómo las recomendaciones particulares pueden afectar el riesgo de ACV o demencia a nivel poblacional en lugar de como se estima en subpoblaciones de alto riesgo. Es importante destacar que, aunque sigue debatiéndose el posible efecto del control de la presión arterial sobre el riesgo de demencia, nuestros hallazgos se alinean con la recomendación del reciente informe de la OMS de que la reducción de la presión arterial tiene beneficios

sustanciales (en términos de riesgo de ACV y mortalidad) que pueden motivar el control de la presión arterial independientemente de sus posibles efectos sobre el riesgo de demencia [44].

REFERENCIAS

1. Larson EB, Langa KM. The rising tide of dementia worldwide. Lancet. 2008;372:430–2.

2. Bauer UE, Briss PA, Goodman RA, Bowman BA. Prevention of chronic disease in the 21st century: Elimination of the leading preventable causes of premature death and disability in the USA. Lancet. 2014;384:45–52. Available from: http://dx.doi.org/10.1016/S0140-6736(14)60648-6

3. Johnson CO, Nguyen M, Roth GA, Nichols E, Alam T, Abate D, et al. Global, regional, and national burden of stroke, 1990–2016: a systematic analysis for the Global Burden of Disease Study 2016. Lancet Neurol. 2019;18:439–58.

4. Livingston G, Huntley J, Sommerlad A, Ames D, Ballard C, Banerjee S, et al. Dementia prevention, intervention, and care: 2020 report of the Lancet Commission. Lancet. 2020;396:413–46.

5. Hughes D, Judge C, Murphy R, Loughlin E, Costello M, Whiteley W, et al. Association of Blood Pressure Lowering with Incident Dementia or Cognitive Impairment: A Systematic Review and Meta-analysis. JAMA - J Am Med Assoc. 2020;323:1934–44.

6. Neal B, MacMahon S, Chapman N, Cutler J, Fagard R, Whelton P, et al. Effects of ACE inhibitors, calcium antagonists, and other blood-pressure-lowering drugs: Results of prospectively designed overviews of randomised trials. Lancet. 2000;356:1955–64.

7. Chobanian A V., Bakris GL, Black HR, Cushman WC, Green LA, Izzo JL, et al. Seventh Report of the Joint National Committee on Prevention, Detection, Evaluation, and Treatment of High Blood Pressure. Hypertension. 2003;42:1206–52.

8. Chiuve SE, Rexrode KM, Spiegelman D, Logroscino G, Manson JE, Rimm EB. Primary prevention of stroke by healthy lifestyle. Circulation. 2008;118:947–54.

9. Braillon A, Larsson SC, Akesson A. Healthy diet and lifestyle and risk of stroke in a prospective cohort of women. Neurology. 2015;84:2293–2293.

10. Zhang Y, Tuomilehto J, Jousilahti P, Wang Y, Antikainen R, Hu G. Lifestyle factors and antihypertensive treatment on the risks of ischemic and hemorrhagic stroke. Hypertension. 2012;60:906–12.

11. Prince MJ, Bird AS, Blizard RA, Mann AH. Is the cognitive function of older patients affected by antihypertensive treatment? Results from 54 months of the Medical Research Council's treatment trial of hypertension in older adults. BMJ. 1996;312:801–5.

12. Forette F, Seux ML, Staessen JA, Thijs L, Birkenhäger WH, Babarskiene MR, et al. Prevention of dementia in randomised double-blind placebo-controlled Systolic Hypertension in Europe (Syst-Eur) trial. Lancet. 1998;352:1347–51.

13. Tzourio C, Anderson C, Chapman N, Woodward M, Neal B, MacMahon S, et al. Effects of blood pressure lowering with perindopril and indapamide therapy on dementia and cognitive decline in patients with cerebrovascular disease. Arch Intern Med. 2003;163:1069–75.

14. Lithell H, Hansson L, Skoog I, Elmfeldt D. The Study on Cognition and Prognosis in the Elderly (SCOPE): principal results of a randomized double-blind intervention trial. J Hypertens. 2003;21:875–86.

15. Diener HC, Sacco RL, Yusuf S, Cotton D, Ôunpuu S, Lawton WA, et al. Effects of aspirin plus extended-release dipyridamole versus clopidogrel and telmisartan on disability and cognitive function after recurrent stroke in patients with ischaemic stroke in the Prevention Regimen for Effectively Avoiding Second Strokes

(PRoFE). Lancet Neurol. 2008;7:875–84.

16. Anderson C, Teo K, Gao P, Arima H, Dans A, Unger T, et al. Renin-angiotensin system blockade and cognitive function in patients at high risk of cardiovascular disease: Analysis of data from the ONTARGET and TRANSCEND studies. Lancet Neurol. 2011;10:43–53.

17. Williamson JD, Pajewski NM, Auchus AP, Bryan RN, Chelune G, Cheung AK, et al. Effect of Intensive vs Standard Blood Pressure Control on Probable Dementia: A Randomized Clinical Trial. JAMA - J Am Med Assoc. 2019;321:553–61.

18. Bosch J, Yusuf S, Pogue J, Sleight P, Lonn E, Rangoonwala B, et al. Use of ramipril in preventing stroke : double blind randomised trial. BMJ. 2002;324:1–5.

19. Ding J, Davis-Plourde KL, Sedaghat S, Tully PJ, Wang W, Phillips C, et al. Antihypertensive medications and risk for incident dementia and Alzheimer's disease: a meta-analysis of individual participant data from prospective cohort studies. Lancet Neurol. 2020;19:61–70.

20. Liang X, Shan Y, Ding D, Zhao Q, Guo Q, Zheng L, et al. Hypertension and high blood pressure are associated with dementia among Chinese dwelling elderly: The Shanghai aging study. Front Neurol. 2018;9:1–7.

21. Zhang Y, Young JG, Thamer M, Hernán MA. Comparing the Effectiveness of Dynamic Treatment Strategies Using Electronic Health Records: An Application of the Parametric g-Formula to Anemia Management Strategies. Health Serv Res. 2018;

22. Taubman SL, Robins JM, Mittleman MA, Hernán MA. Intervening on risk factors for coronary heart disease: An application of the parametric g-formula. Int J Epidemiol. 2009;38:1599–611.

23. Jain P, Danaei G, Robins JM, Manson JAE, Hernán MA. Smoking cessation and long-term weight gain in the Framingham Heart Study: an application of the parametric g-formula for a continuous outcome. Eur J Epidemiol. 2016;

24. Danaei G, García Rodríguez LA, Cantero OF, Logan RW, Hernán MA. Electronic medical records can be used to emulate target trials of sustained treatment strategies. J Clin Epidemiol. 2018;96:12–22.

25. Garcia-Aymerich J, Varraso R, Danaei G, Camargo CA, Hernán MA. Incidence of adult-onset asthma after hypothetical interventions on body mass index and physical activity. Web material. Am J Epidemiol. 2014;

26. Hernán MA, Robins JM. Causal Inference. Boca Raton: Chapman & Hall/CRC, forthcoming.; 2019.

27. Lodi S, Phillips A, Lundgren J, Logan R, Sharma S, Cole SR, et al. Effect Estimates in Randomized Trials and Observational Studies: Comparing Apples with Apples. Am J Epidemiol. 2019;188:1569–77.

28. Hernán MA, Alonso A, Logan R, Grodstein F, Michels KB, Willett WC, et al. Observational studies analyzed like randomized experiments: An application to postmenopausal hormone therapy and coronary heart disease. Epidemiology. 2008;19:766–79.

29. Ikram MA, Brusselle GGO, Murad SD, van Duijn CM, Franco OH, Goedegebure A, et al. The Rotterdam Study: 2018 update on objectives, design and main results. Eur J Epidemiol. 2017;

30. Wieberdink RG, Ikram MA, Hofman A, Koudstaal PJ, Breteler MMB. Trends in stroke incidence rates and stroke risk factors in Rotterdam, the Netherlands from 1990 to 2008. Eur J Epidemiol. 2012;27:287–95.

31. Akoudad S, Portegies MLP, Koudstaal PJ, Hofman A, Van Der Lugt A, Ikram MA, et al. Cerebral Microbleeds Are Associated with an Increased Risk of Stroke: The Rotterdam Study. Circulation. 2015;132:509–16.

32. de Bruijn RFAG, Bos MJ, Portegies MLP, Hofman A, Franco OH, Koudstaal PJ, et al. The potential for prevention of dementia across two decades: The prospective, population-based Rotterdam Study. BMC Med. 2015;13:1–8.

33. Ott A, van Rossum CTM, van Harskamp F, van de Mheen H, Hofman A, Breteler MMB. Education and the incidence of dementia in a large population-based study: The Rotterdam Study. Neurology.1999;52:663 LP – 663.

34. Danaei G, Pan A, Hu FB, Hernán MA. Hypothetical midlife interventions in women and risk of type 2 diabetes. Epidemiology. 2013;24:122–8.

35. Vangen-Lønne AM, Ueda P, Gulayin P, Wilsgaard T, Mathiesen EB, Danaei G. Hypothetical interventions to prevent stroke: an application of the parametric g-formula to a healthy middle-aged population. Eur J Epidemiol. 2018;

36. Dickerman BA, Giovannucci E, Pernar CH, Mucci LA, Hernán MA, Chan HT, et al. Guideline-Based Physical Activity and Survival Among US Men With Nonmetastatic Prostate Cancer. Am J Epidemiol. 2018;188:579–86.

37. Young JG, Stensrud MJ, Tchetgen EJT, Hernán MA. A causal framework for classical statistical estimands in failure time settings with competing events. Stat Med. 2020;1:1–38.

38. Ettehad D, Emdin CA, Kiran A, Anderson SG, Callender T, Emberson J, et al. Blood pressure lowering for prevention of cardiovascular disease and death: A systematic review and meta-analysis. Lancet. 2016;387:957–67.

39. Walker KA, Sharrett AR, Wu A, Schneider ALC, Albert M, Lutsey PL, et al. Association of midlife to late-life blood pressure patterns with incident dementia. JAMA - J Am Med Assoc. 2019;322:535–45.

40. Beam CR, Kaneshiro C, Jang JY, Reynolds CA, Pedersen NL, Gatz M. Differences between Women and Men in Incidence Rates of Dementia and Alzheimer's Disease. J Alzheimer's Dis. 2018;64:1077–83.

41. Joe E, Ringman JM. Cognitive symptoms of Alzheimer's disease: clinical management and prevention. BMJ. 2019;367:l6217.

42. Hernán MA. Does water kill? A call for less casual causal inferences. Ann Epidemiol. 2016;26:674–80.

43. Hernán MA, Vanderweele TJ. Compound treatments and transportability of causal inference. Epidemiology. 2011;22:368–77.

44. World Health Organization. Risk reduction of cognitive decline and dementia: WHO guidelines. WHO. 2019. Available from: https://www.who.int/mental_health/neurology/dementia/risk_reduction_gdg_meeting/en/

Figura 1. Diagrama de flujo

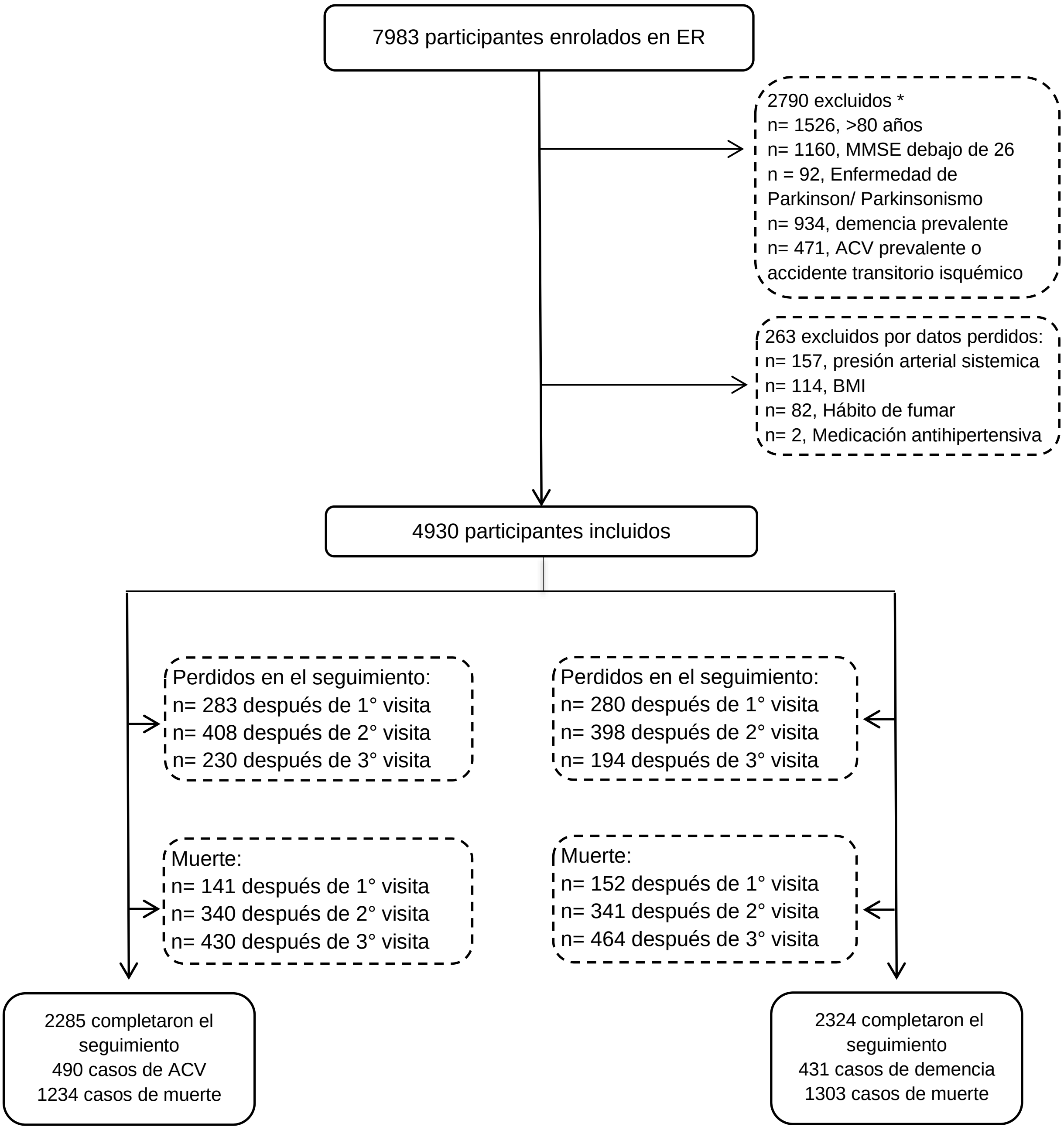

Figura 2. Riesgo de ACV bajo el curso natural y bajo la intervención combinada: Reducir la PAS en 20% si es mayor a 140 mmHg y dejar de fumar durante 15 años de seguimiento

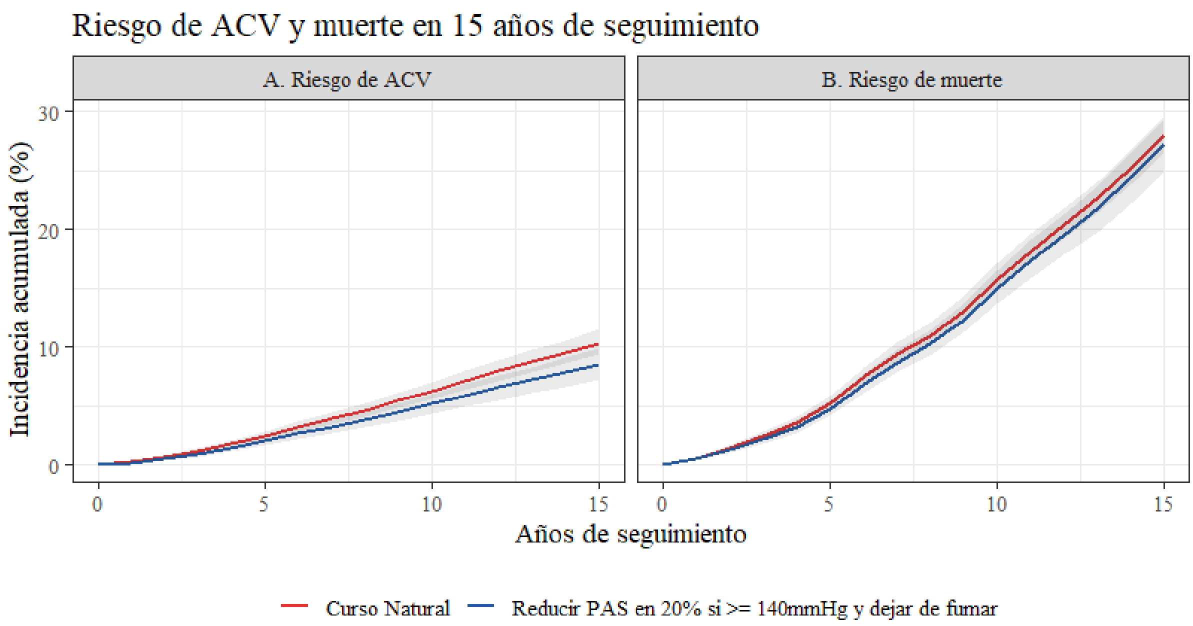

Figura 3. Riesgo de demencia bajo el curso natural y bajo la intervención combinada: Reducir la PAS en 20% si es mayor a 140 mmHg y dejar de fumar durante 15 años de seguimiento

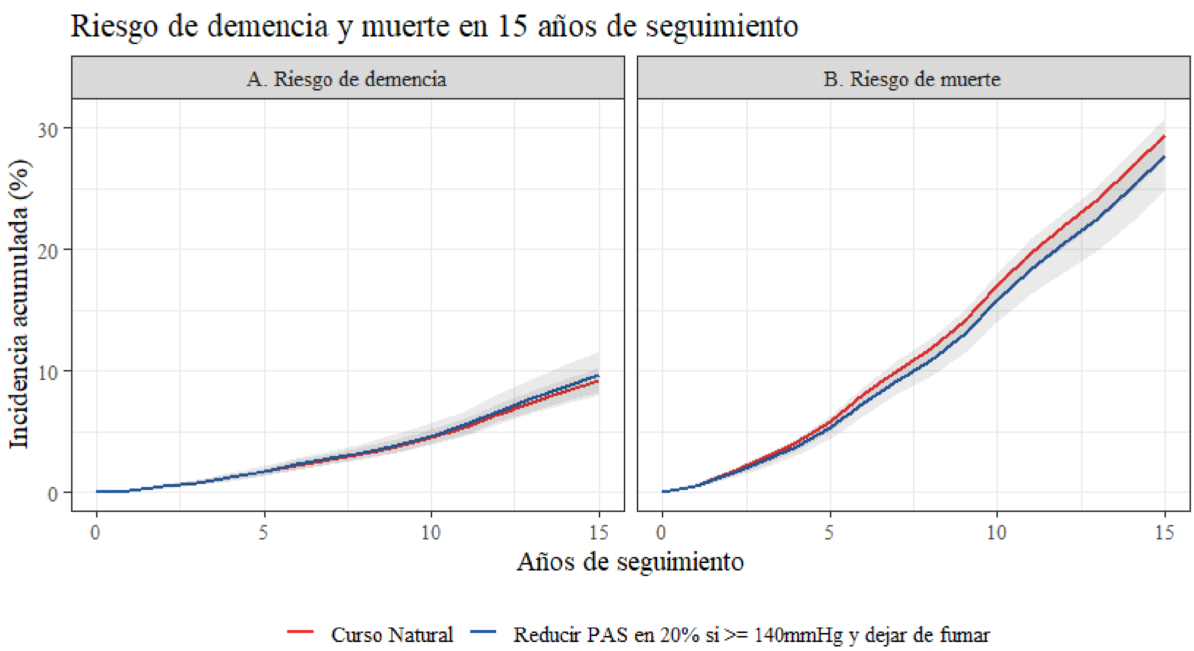

Tabla 1. Características basales de la cohorte (n=4930)

	**Total**
Mujer, n (%)	2824 (57.3)
Edad, media (DE) (años)	66.2 (6.6)
Gen Apoe4, n (%)	
No portador	3388 (68.7)
Portador	1322 (26.8)
No disponible	220 (4.5)
Nivel de educación, n (%)	
Primaria	2378 (48.6)
Secundaria	2044 (41.8)
Superior	472 (9.6)
MMSE, media (DE)	28.2 (1.2)
BMI, media (DE) (kg/m2)	26.3 (3.6)
Consumo de cigarrillo, n (%)	
Nunca fumó	1551 (31.5)
Fumó previamente	2193 (44.5)
Fuma actualmente	1186 (24.1)
Ingesta de alcohol, media (DE) (g/día)	10.7 (15.3)
Presión arterial sistólica, media (DE) (mmHg)	137.3 (21.5)
Colesterol total, media (DE) (mmol/dl)	6.7 (1.2)
HDL, mean (SD) (mmol/dl)	1.4 (0.4)
Hipertensión prevalente, n (%)	2789 (56.6)
Medicación antihipertensiva, n (%)	1360 (27.6)
Enfermedad cardiaca prevalente, n (%)	368 (7.7)
Cancer prevalente, n (%)	20 (0.4)
Diabetes prevalente, n (%)	432 (12.9)

MMSE: Mini Mental State Examination

BMI: Indice de masa corporal

HDL: Lipoproteinas de alta densidad

Tabla 2. Riesgo de ACV en 15 años de seguimiento bajo el curso natural e intervenciones hipotéticas

No	Intervención	Riesgo absoluto (95% CI)	Riesgo relativo (95% CI)	Diferencia de riesgo (95% CI)	Total intervenidos (%)
0	Curso natural	10.3 (9.3, 11.5)	1 (1 to 1)	0 (0 to 0)	0.00
1	Mantener la PAS debajo de 120 mmHg	9 (7.5, 10.7)	0.87 (0.74, 1.02)	-1.3 (-2.8, 0.2)	97.8
2	Mantener la PAS debajo de 140 mmHg	9.3 (8.2, 10.6)	0.9 (0.83, 0.98)	-1 (-1.8, -0.2)	83.5
3	Reducir la PAS en 10% si es mayor a 140 mmHg	9.3 (8, 10.6)	0.9 (0.8, 0.98)	-1.1 (-2.1, -0.2)	82.7
4	Reducir la PAS en 20% si es mayor a 140 mmHg	9.2 (7.8, 10.6)	0.89 (0.76, 1)	-1.1 (-2.5, 0)	82.7
5	Dejar de fumar	9.6 (8.6, 10.8)	0.93 (0.89, 0.97)	-0.7 (-1.2, -0.3)	25.9
6	Intervención conjunta 1 + 5	8.3 (6.9, 10)	0.81 (0.68, 0.95)	-2 (-3.4, -0.5)	98.7
7	Intervención conjunta 2 + 5	8.8 (7.6, 9.9)	0.85 (0.76, 0.92)	-1.6 (-2.6, -0.8)	88.6
8	Intervención conjunta 3 + 5	8.6 (7.4, 9.8)	0.83 (0.74, 0.92)	-1.7 (-2.8, -0.8)	88.2
9	Intervención conjunta 4 + 5	8.5 (7.2, 9.9)	0.83 (0.71, 0.94)	-1.8 (-3.1, -0.6)	88.2

PAS: Presión arterial sistólica (mmHg).
Los estimados se calcularon utilizando la g-formula paramétrica contemplando las variables fijas: edad, sexo, educación, PAS, historia de diabetes y enfermedad cardíaca; y covariables que cambian en el tiempo como ser: el proceso de las visitas, hábito de fumar, PAS, BMI, medicación, colesterol total y diagnóstico de diabetes, enfermedad cardiaca, enfermedad de Parkinson, Parkinsonismo, accidente isquémico transitorio, cáncer o demencia

Tabla 3. Riesgo de demencia en 15 años de seguimiento bajo el curso natural e intervenciones hipotéticas

No	Intervención	Riesgo absoluto (95% CI)	Riesgo relativo (95% CI)	Diferencia de riesgo (95% CI)	Total intervenidos (%)
0	Curso natural	9.2 (8.2, 10.3)	1 (1, 1)	0 (0, 0)	0.0
1	Mantener la PAS debajo de 120 mmHg	9.7 (8, 11.9)	1.06 (0.9, 1.24)	0.6 (-0.9, 2.2)	98.2
2	Mantener la PAS debajo de 140 mmHg	9.2 (8, 10.7)	1.01 (0.92, 1.09)	0.1 (-0.7, 0.8)	83.0
3	Reducir la PAS en 10% si es mayor a 140 mmHg	9.2 (8, 10.9)	1.01 (0.92, 1.11)	0 (-0.8, 1)	83.3
4	Reducir la PAS en 20% si es mayor a 140 mmHg	9.5 (8, 11.4)	1.04 (0.91, 1.18)	0.3 (-0.8, 1.7)	83.3
5	Dejar de fumar	9.3 (8.4, 10.6)	1.01 (0.98, 1.06)	0.1 (-0.2, 0.5)	25.9
6	Intervención conjunta 1 + 5	9.9 (8, 12.2)	1.08 (0.92, 1.26)	0.8 (-0.8, 2.4)	98.8
7	Intervención conjunta 2 + 5	9.3 (8.1, 10.9)	1.02 (0.93, 1.12)	0.2 (-0.6, 1.1)	88.2
8	Intervención conjunta 3 + 5	9.3 (8, 11.1)	1.02 (0.93, 1.14)	0.2 (-0.7, 1.3)	88.6
9	Intervención conjunta 4 + 5	9.6 (8, 11.6)	1.05 (0.92, 1.2)	0.4 (-0.7, 2)	88.6

PAS: Presión arterial sistólica (mmHg).
Los estimados se calcularon utilizando la g-formula paramétrica contemplando las variables fijas: edad, sexo, educación, PAS, historia de diabetes y enfermedad cardíaca; y covariables que cambian en el tiempo como ser: el proceso de las visitas, hábito de fumar, PAS, BMI, medicación, colesterol total y diagnóstico de diabetes, enfermedad cardiaca, enfermedad de Parkinson, parkinsonismo, accidente isquémico transitorio, cáncer o demencia

Table 4. Efecto de una intervención conjunta: Reducir la PAS en 20% si es mayor a 140 mmHg y dejar de fumar, en el riesgo de ACV y demencia por subgrupos

	ACV				Demencia			
**Subgrupo**	**Riesgo bajo el curso natural (IC95%)**	**Riesgo bajo la intervención conjunta (IC95%)**	**Riesgo relative (IC95%)**	**Diferencia de riesgos (IC95%)**	**Riesgo bajo el curso natural (IC95%)**	**Riesgo bajo la intervención conjunta (IC95%)**	**Riesgo relative (IC95%)**	**Diferencia de riesgos (IC95%)**
Cohorte completa	10.3 (9.3, 11.5)	8.5 (7.2, 9.9)	0.83 (0.71, 0.94)	-1.8 (-3.1, -0.6)	9.2 (8.2, 10.3)	9.6 (8, 11.6)	1.05 (0.92, 1.2)	0.4 (-0.7, 2)
Edad debajo de 65 años (n=2303)	6.3 (5, 7.9)	4.7 (3.3, 6.4)	0.75 (0.56, 0.98)	-1.6 (-2.7, -0.2)	4 (3.2, 5.7)	4.2 (3, 6.1)	1.04 (0.73, 1.37)	0.2 (-1, 1.4)
Edad entre 65 y 80 años (n = 2627)	14 (12.6, 15.8)	11.9 (9.6, 14.5)	0.85 (0.71, 1)	-2.2 (-4.3, 0)	13.8 (12.4, 15.6)	14.2 (12, 17.3)	1.03 (0.87, 1.2)	0.4 (-1.8, 2.9)
Mujeres (n = 2824)	9.3 (8.1, 10.9)	7.7 (6.1, 10.3)	0.83 (0.71, 1.02)	-1.6 (-2.9, 0.3)	11.2 (9.7, 12.7)	10.1 (8.4, 12.3)	0.91 (0.73, 1.05)	-1.1 (-3.4, 0.5)
Hombres (n = 2106)	11.6 (10.2, 13.5)	9.4 (7.5, 12.3)	0.81 (0.67, 1.01)	-2.2 (-4, 0.1)	7 (6, 8.9)	9.8 (7.2, 13)	1.4 (1.12, 1.64)	2.8 (0.8, 4.5)
Sin medicación hipertensiva al inicio del estudio (n = 3570)	9 (8.1, 10.6)	7 (5.8, 9.1)	0.78 (0.66, 0.94)	-2 (-3.3, -0.5)	8.8 (8, 10.2)	9.9 (7.7, 11.8)	1.12 (0.95, 1.27)	1.1 (-0.4, 2.4)
Sin historia de enfermedad cardíaca al inicio del estudio (n = 4406)	9.9 (9.1, 10.9)	8.2 (6.8, 10.1)	0.83 (0.7, 0.99)	-1.6 (-2.9, -0.1)	8.8 (8, 10)	9.2 (7.6, 11.1)	1.05 (0.9, 1.16)	0.4 (-0.9, 1.6)

PAS: Presión arterial sistólica (mmHg).
Los estimados se calcularon utilizando la g-formula paramétrica contemplando las variables fijas: edad, sexo, educación, PAS, historia de diabetes y enfermedad cardíaca; y covariables que cambian en el tiempo como ser: el proceso de las visitas, hábito de fumar, PAS, BMI, medicación, colesterol total y diagnóstico de diabetes, enfermedad cardiaca, enfermedad de Parkinson, parkinsonismo, accidente isquémico transitorio, cáncer o demencia
